# Supplementary material for: Recommendations to address uncertainties in environmental risk assessment using toxicokinetic-toxicodynamic models
Source: Sci Rep. 2019 Aug 7;9:11432. doi: 10.1038/s41598-019-47698-0 (PMC6685980; doi:10.1038/s41598-019-47698-0)
Supplement: Supplementary file 1 — Supplementary Material - Simulations [file 41598_2019_47698_MOESM1_ESM.pdf]

Supplementary Material - Simulations

Recommendations to address uncertainties in environmental risk assessment using toxicokinetic-toxicodynamic models

Virgile Baudrot[1,2,*] **and Sandrine Charles**[1]

[1]Univ Lyon, Université Lyon 1, UMR CNRS 5558, Laboratoire de Biométrie et Biologie Évolutive, F-69100 Villeurbanne, France

[2] INRA, BioSP, 84000 Avignon, France

Corresponding author 1 Virgile Baudrot
Mail: virgile.baudrot@posteo.net

Corresponding author 2 Sandrine Charles
Mail: sandrine.charles@univ-lyon1.fr

Supplementary Material - Simulations

Virgile Baudrot and Sandrine Charles

- Results of fit
  - Fitting plot
  - PPC: Posterior Predictive Check
  - Posteriors vs. Priors
  - Pairs plots
- LCx
  - LCx at final time
  - LCx along Time
  - LCx along X
- MFx
  - MFx along Time
  - MFx along X
- Predictions
  - Depuration times for every compounds
  - Prediction with different pulses frequencies and amplitudes

Results of fit

Fitting plot

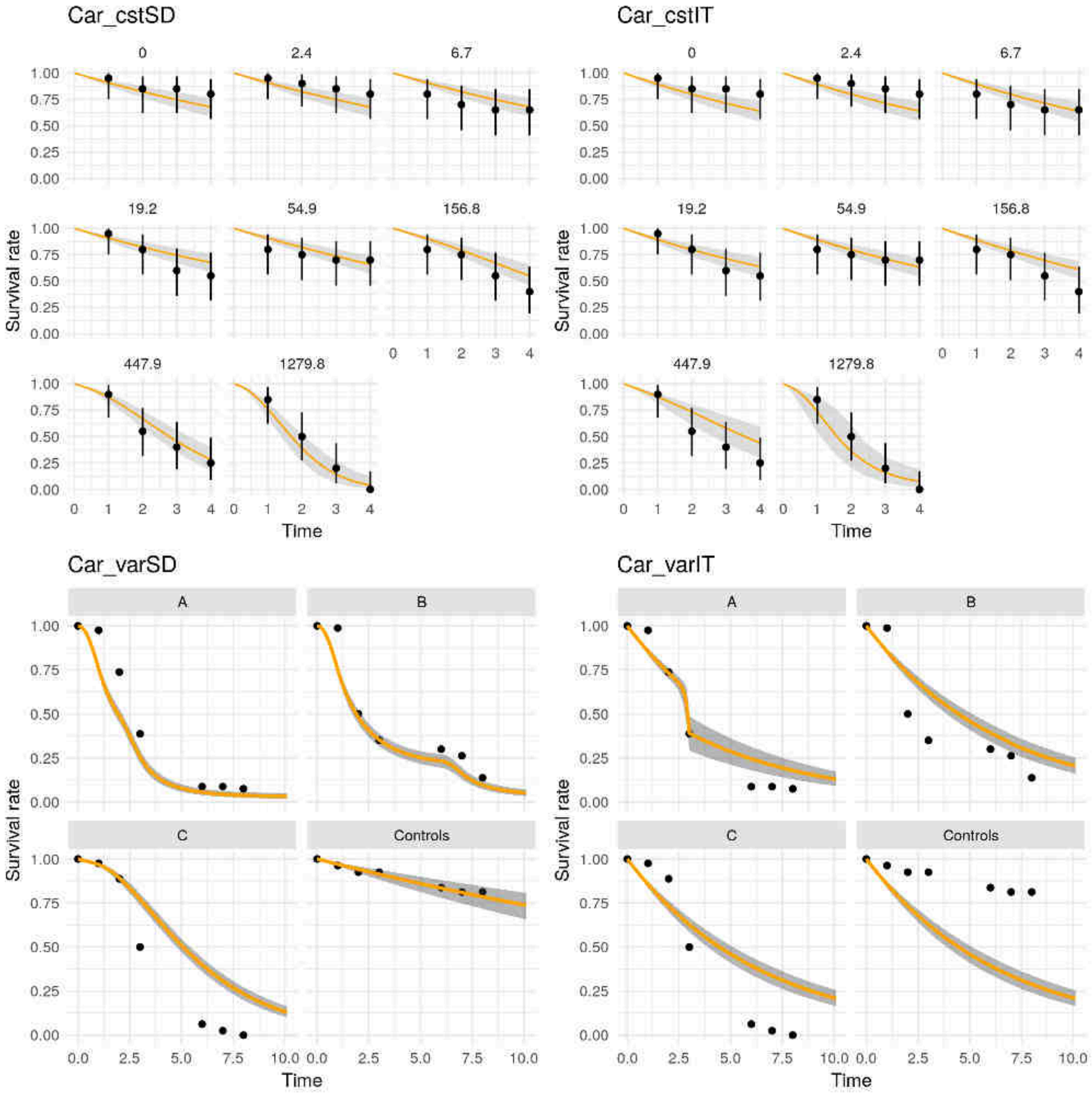

PPC: Posterior Predictive Check

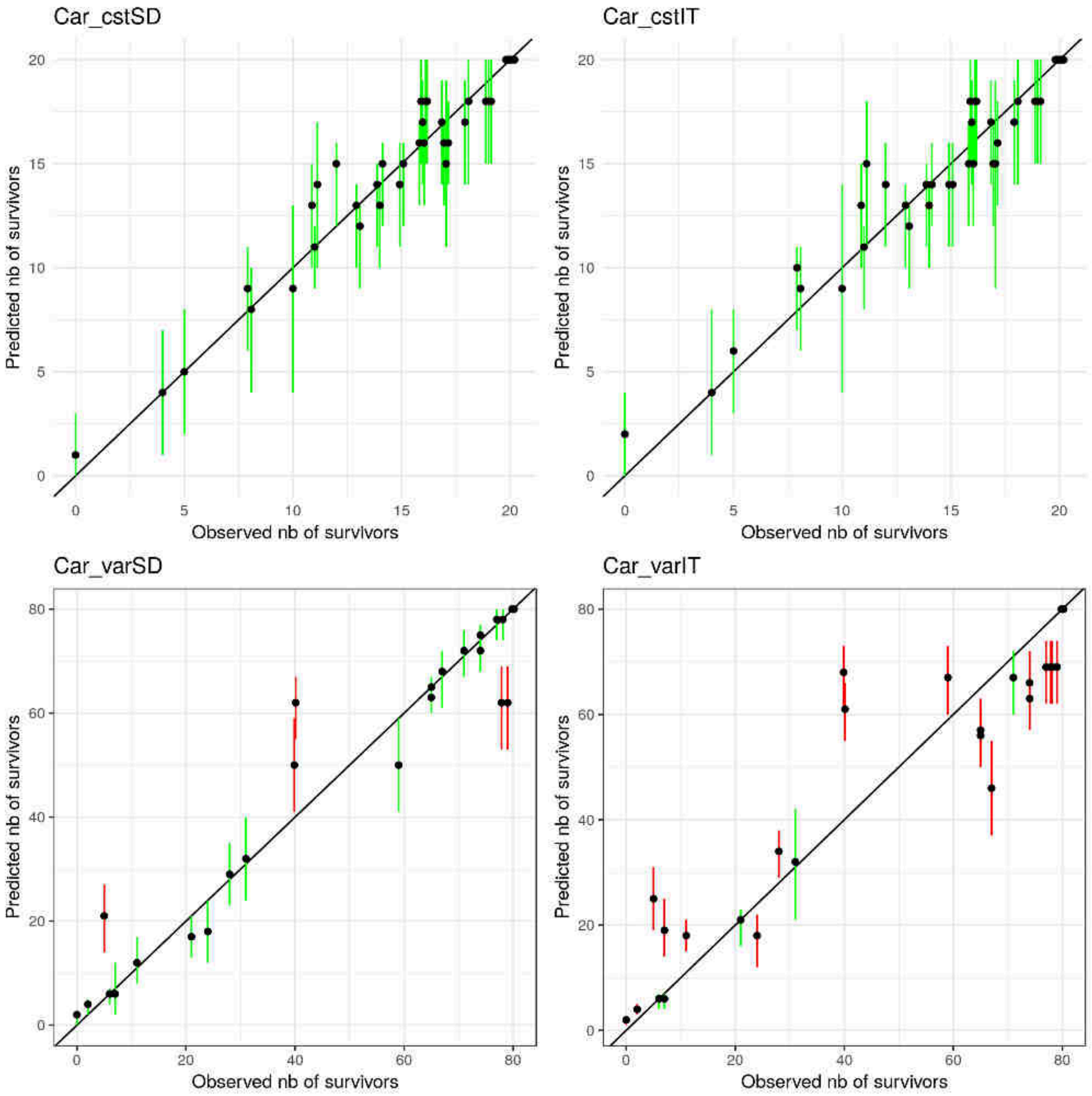

Posteriors vs. Priors

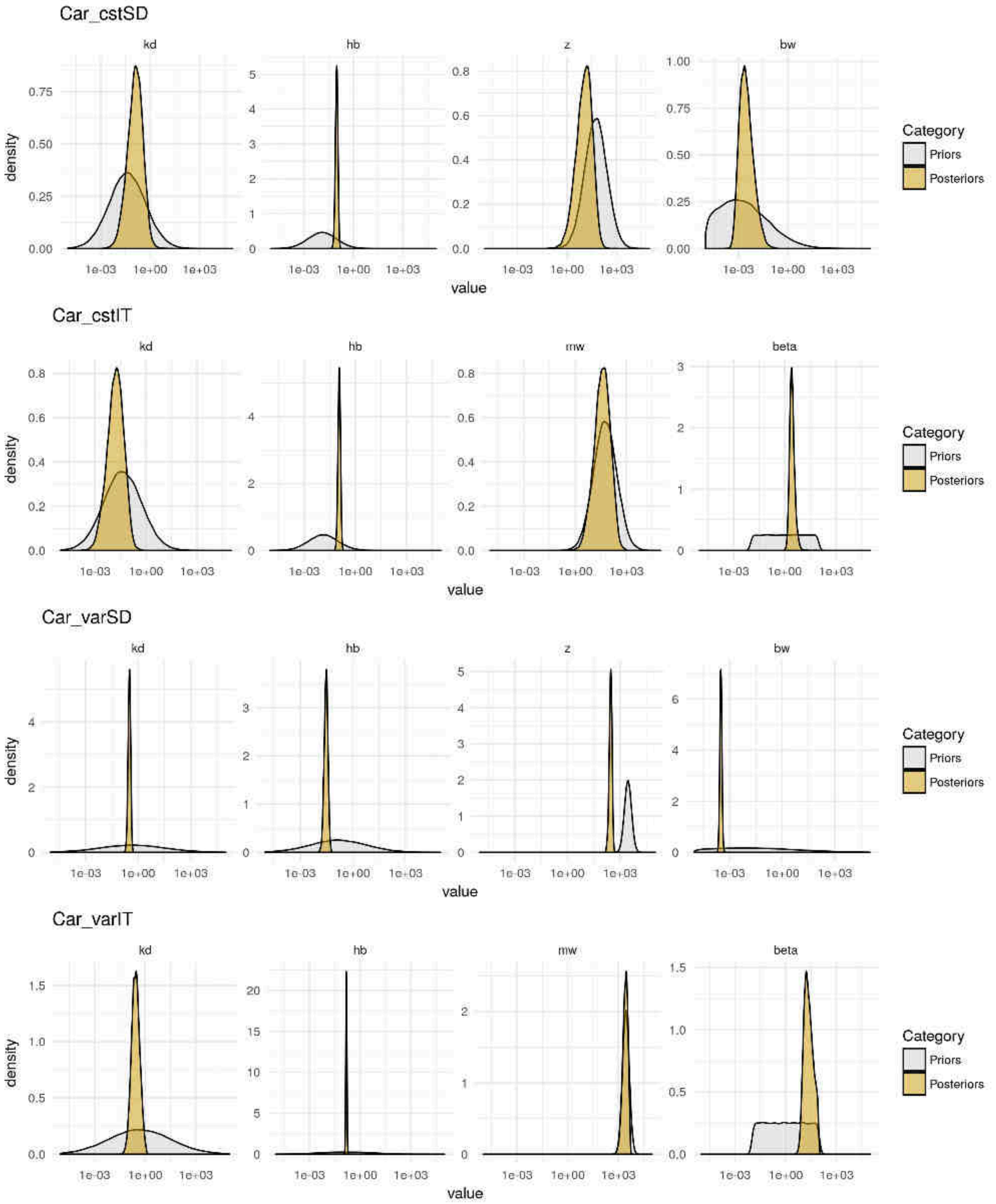

Pairs plots

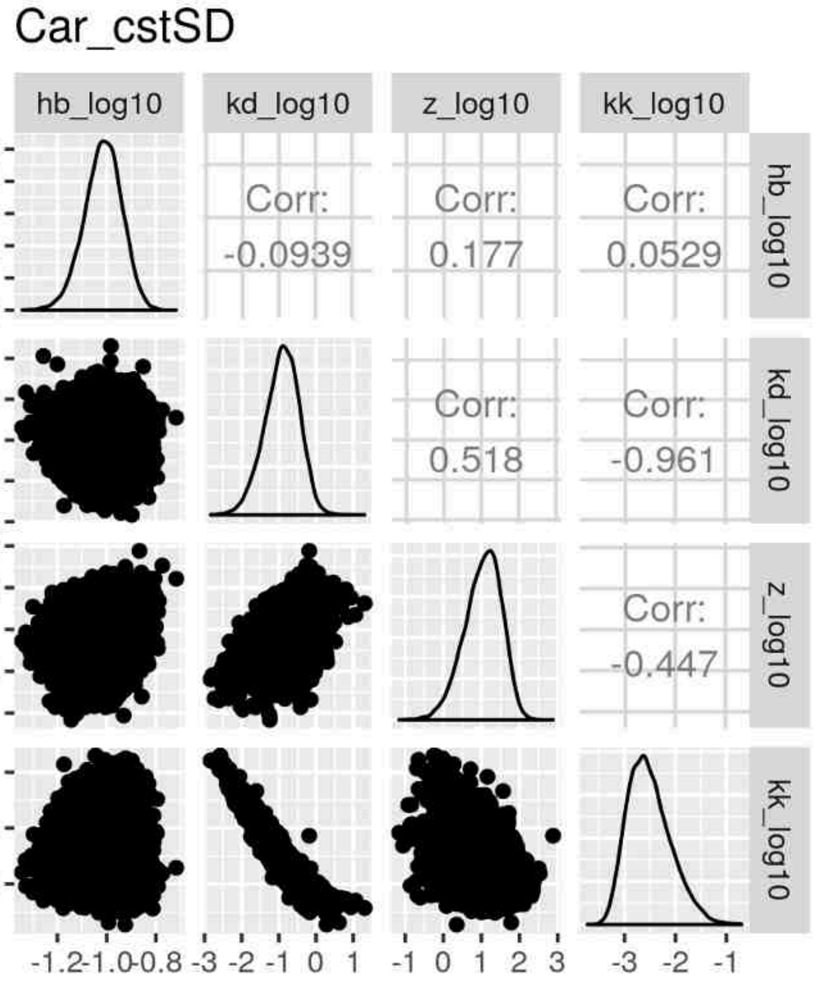

Car_cstSD
hb_log10
kd_log10
z_log10
kk_log10
Corr:
-0.0939
Corr:
0.177
Corr:
0.0529
Corr:
0.518
Corr:
-0.961
Corr:
-0.447

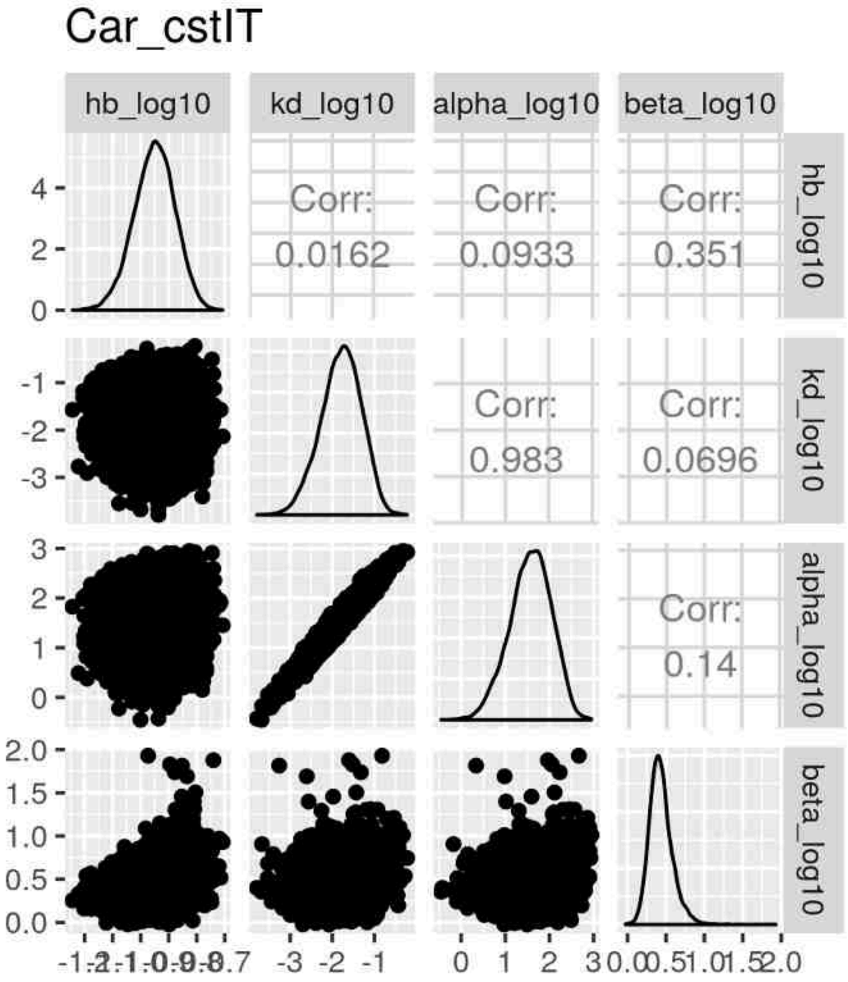
Car_cstIT
hb_log10
kd_log10
alpha_log10
beta_log10
Corr:
0.0162
Corr:
0.0933
Corr:
0.351
Corr:
0.983
Corr:
0.0696
Corr:
0.14

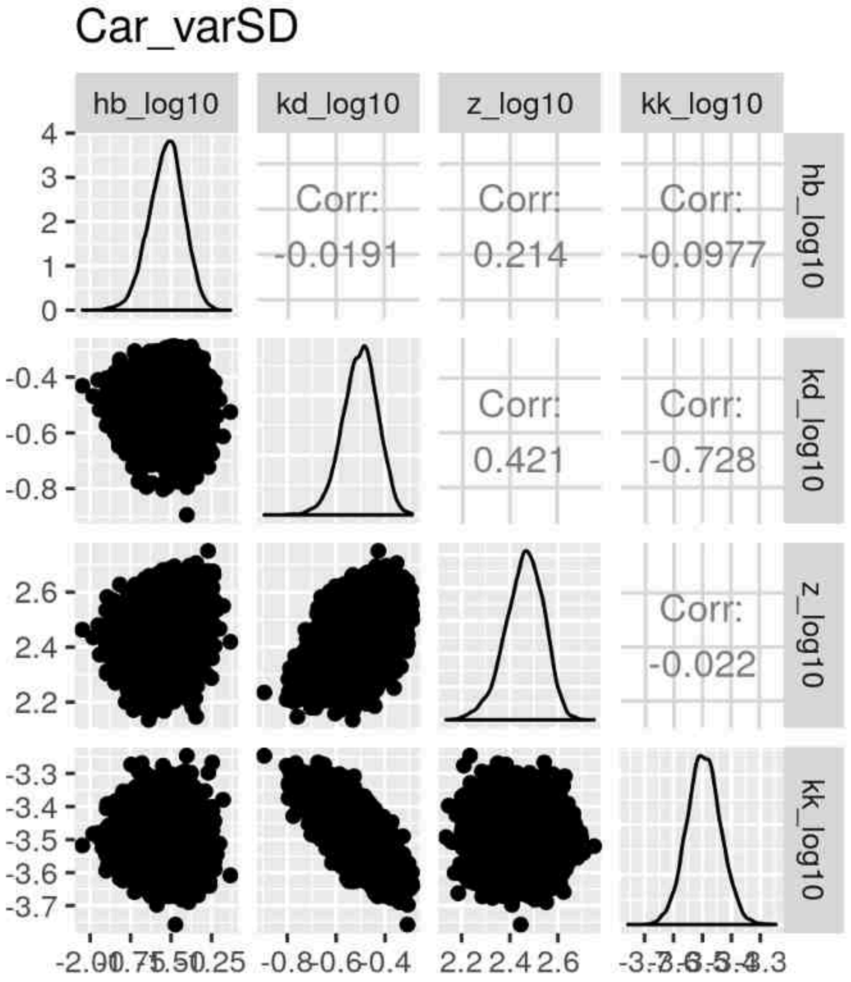

Car_varSD
hb_log10
kd_log10
z_log10
kk_log10
Corr:
-0.0191
Corr:
0.214
Corr:
-0.0977
Corr:
0.421
Corr:
-0.728
Corr:
-0.022

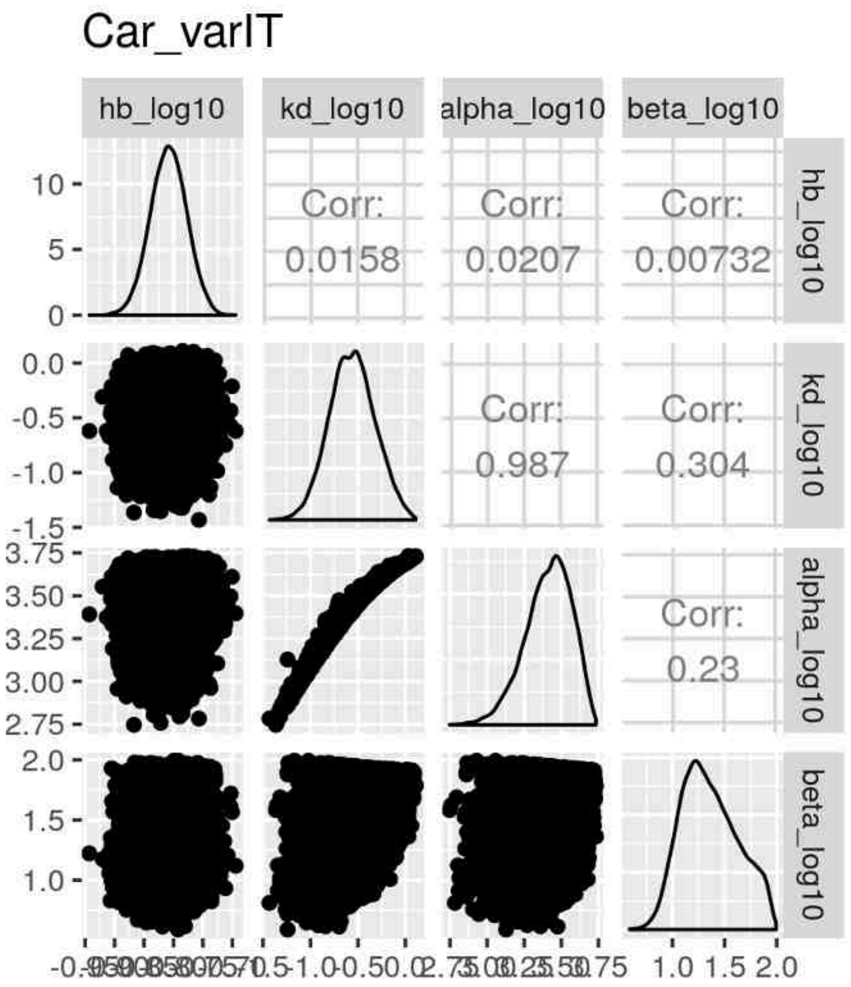

Car_varIT
hb_log10
kd_log10
alpha_log10
beta_log10
Corr:
0.0158
Corr:
0.0207
Corr:
0.00732
Corr:
0.987
Corr:
0.304
Corr:
0.23

LCx

LCx at final time

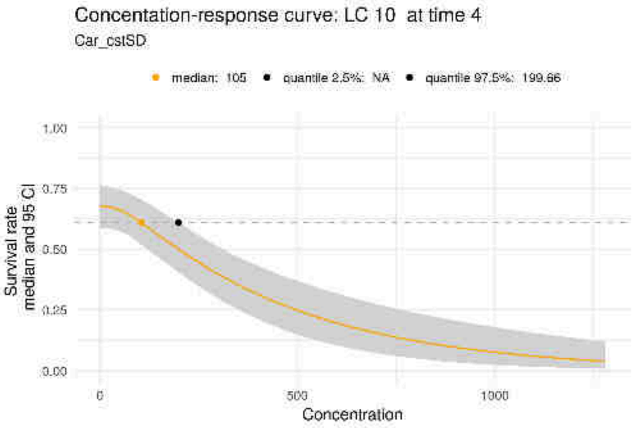

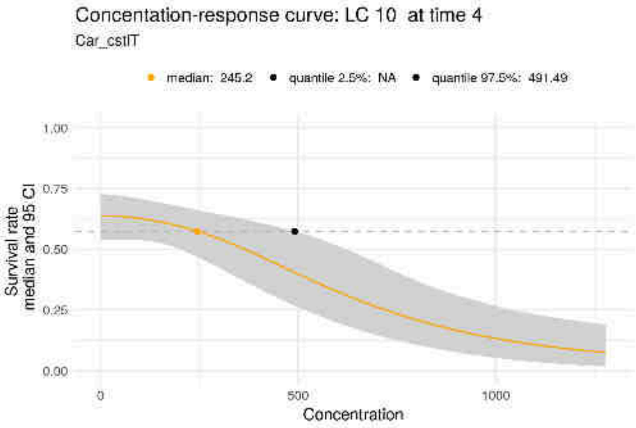

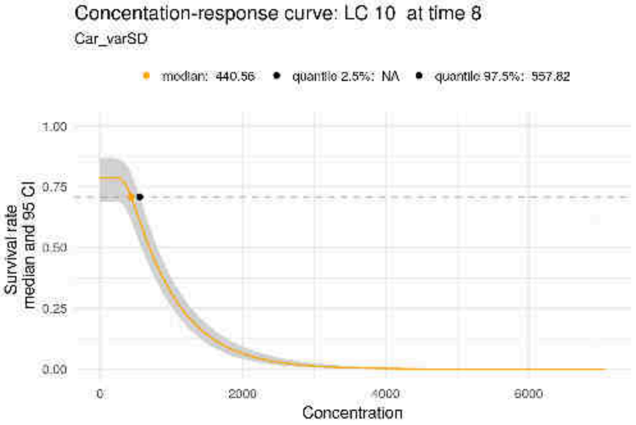

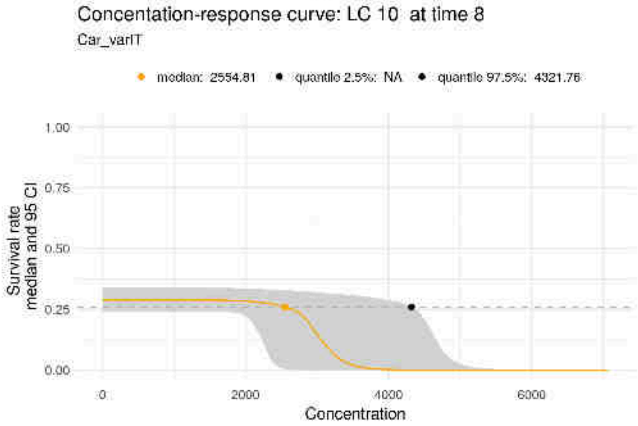

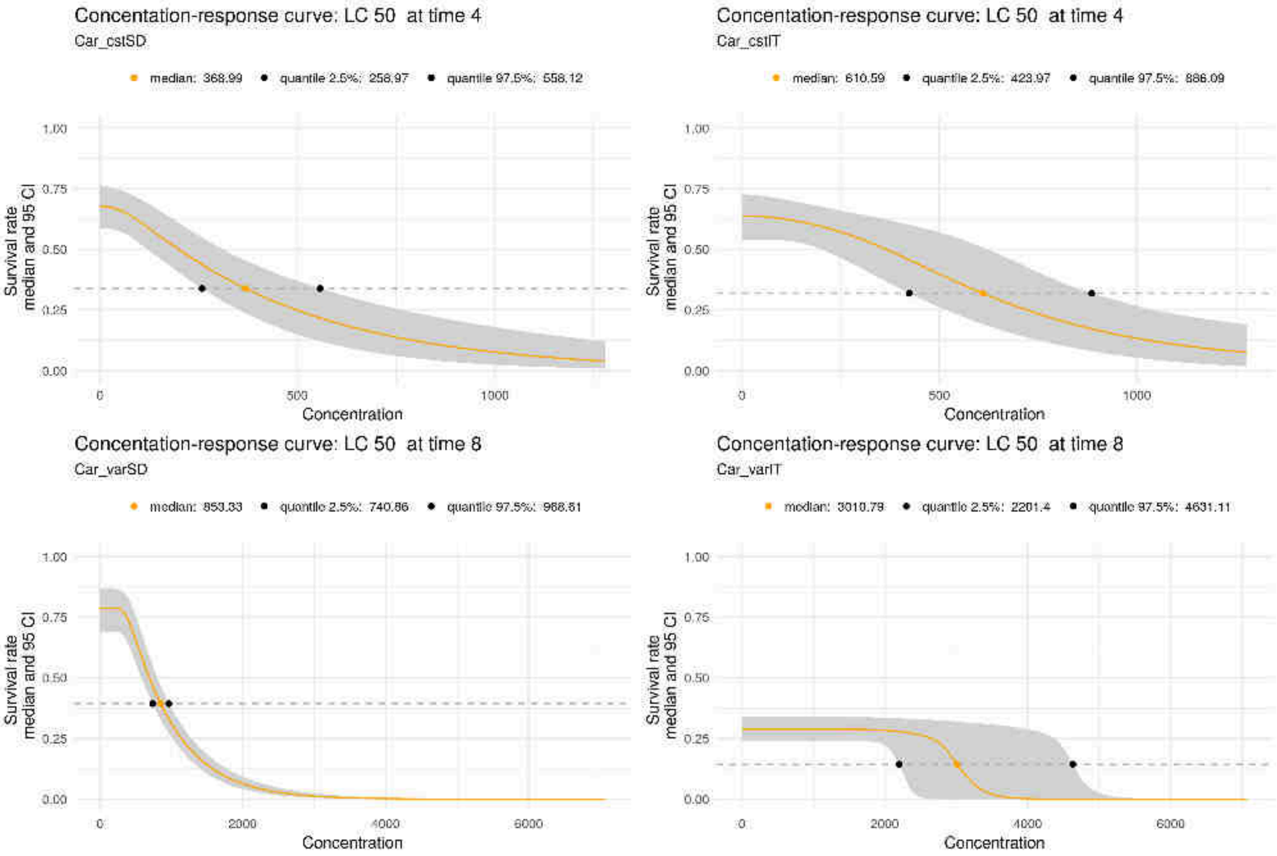
Concentation-response curve: LC 50 at time 4
Car_cstSD
median: 368.99
quantile 2.5%: 258.97
quantile 97.5%: 558.12
Survival rate
median and 95 CI
1.00
0.75
0.50
0.25
0.00
0
500
1000
Concentration
Concentation-response curve: LC 50 at time 4
Car_cstIT
median: 610.59
quantile 2.5%: 423.97
quantile 97.5%: 886.09
Survival rate
median and 95 CI
1.00
0.75
0.50
0.25
0.00
0
500
1000
Concentration
Concentation-response curve: LC 50 at time 8
Car_varSD
median: 853.33
quantile 2.5%: 740.86
quantile 97.5%: 968.81
Survival rate
median and 95 CI
1.00
0.75
0.50
0.25
0.00
0
2000
4000
6000
Concentration
Concentation-response curve: LC 50 at time 8
Car_varIT
median: 3010.79
quantile 2.5%: 2201.4
quantile 97.5%: 4631.11
Survival rate
median and 95 CI
1.00
0.75
0.50
0.25
0.00
0
2000
4000
6000
Concentration

LCx along Time

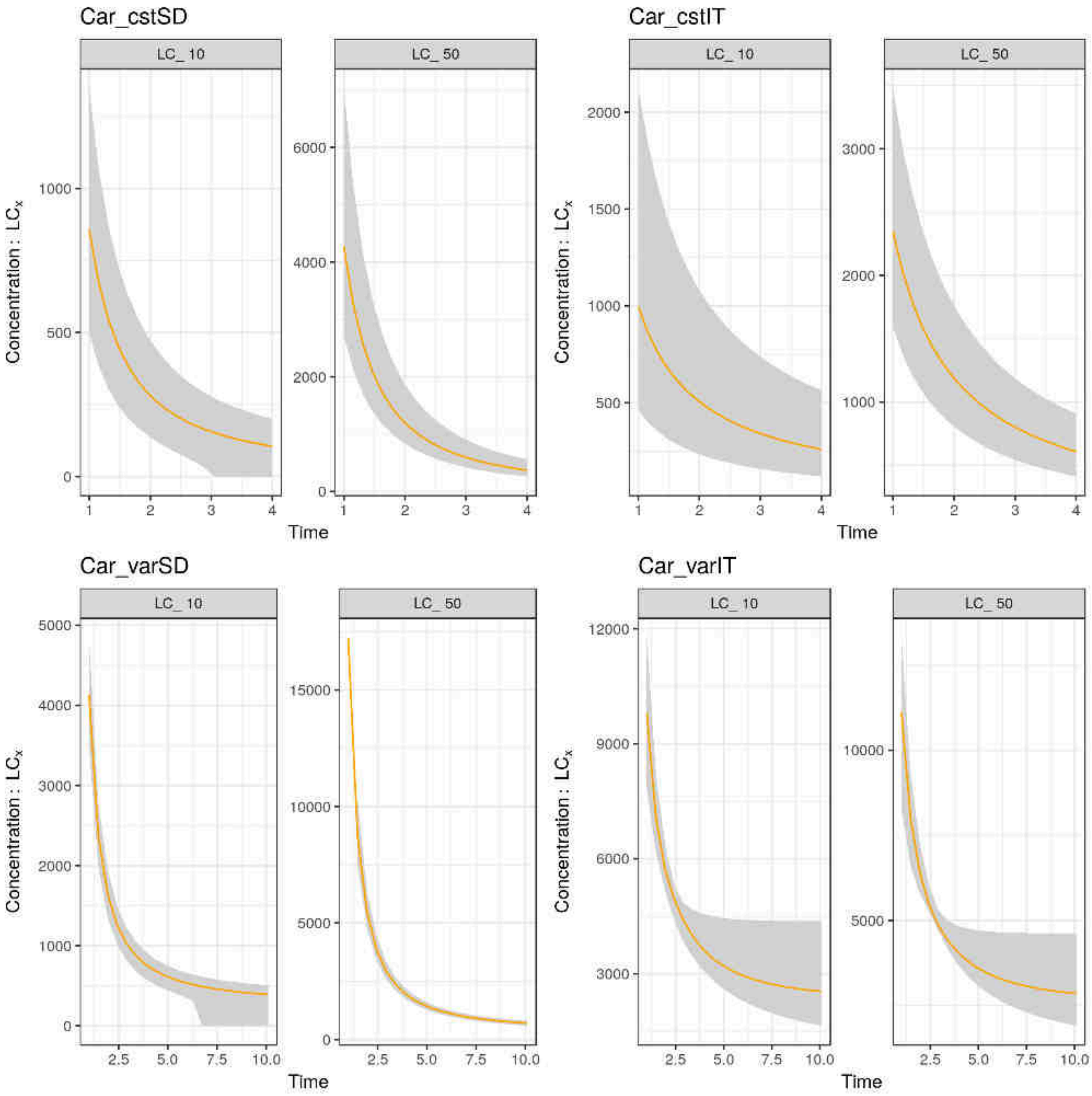

LCx along X

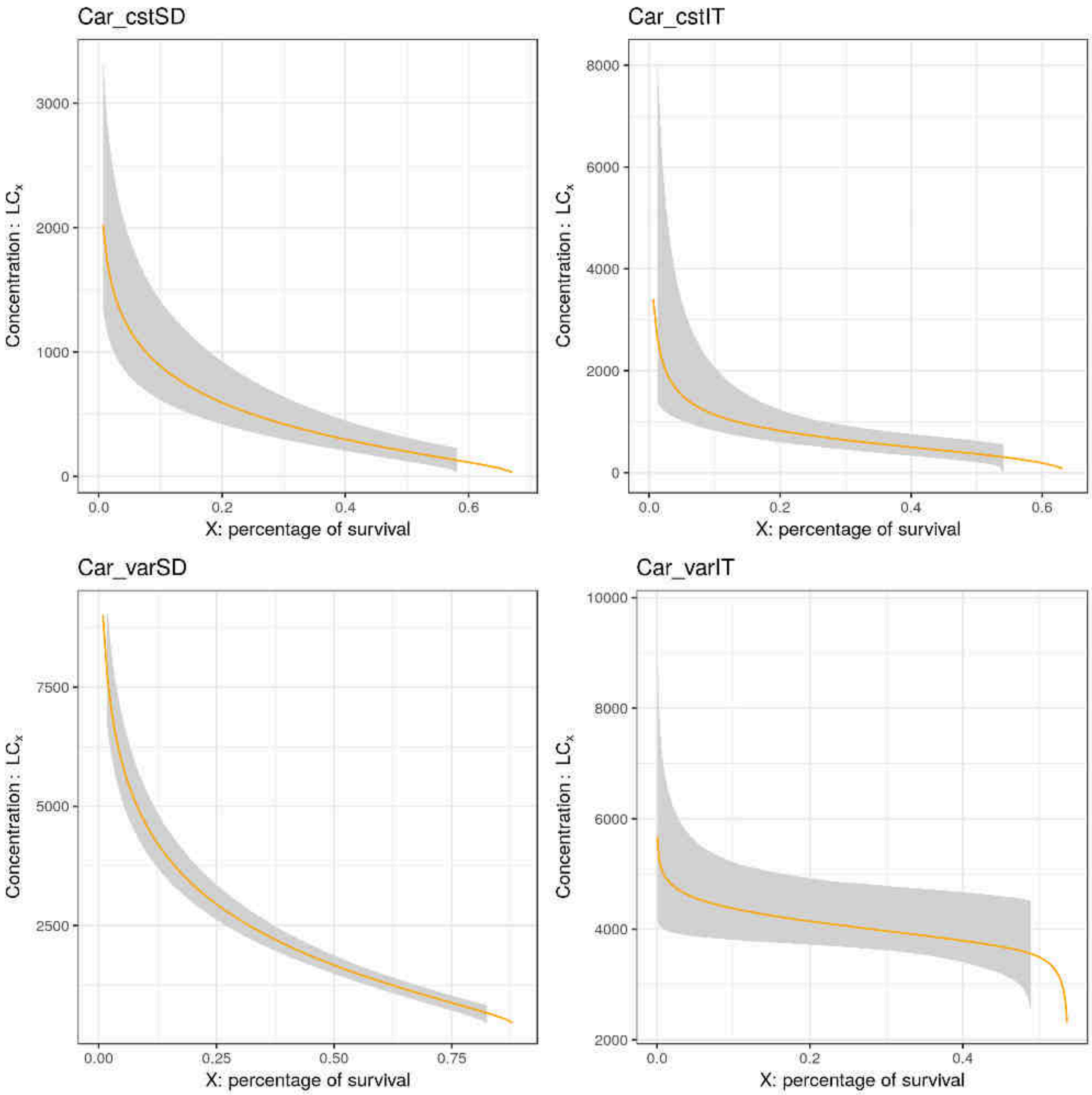

MFx

MFx along Time

GUTS-SD GUTS-IT

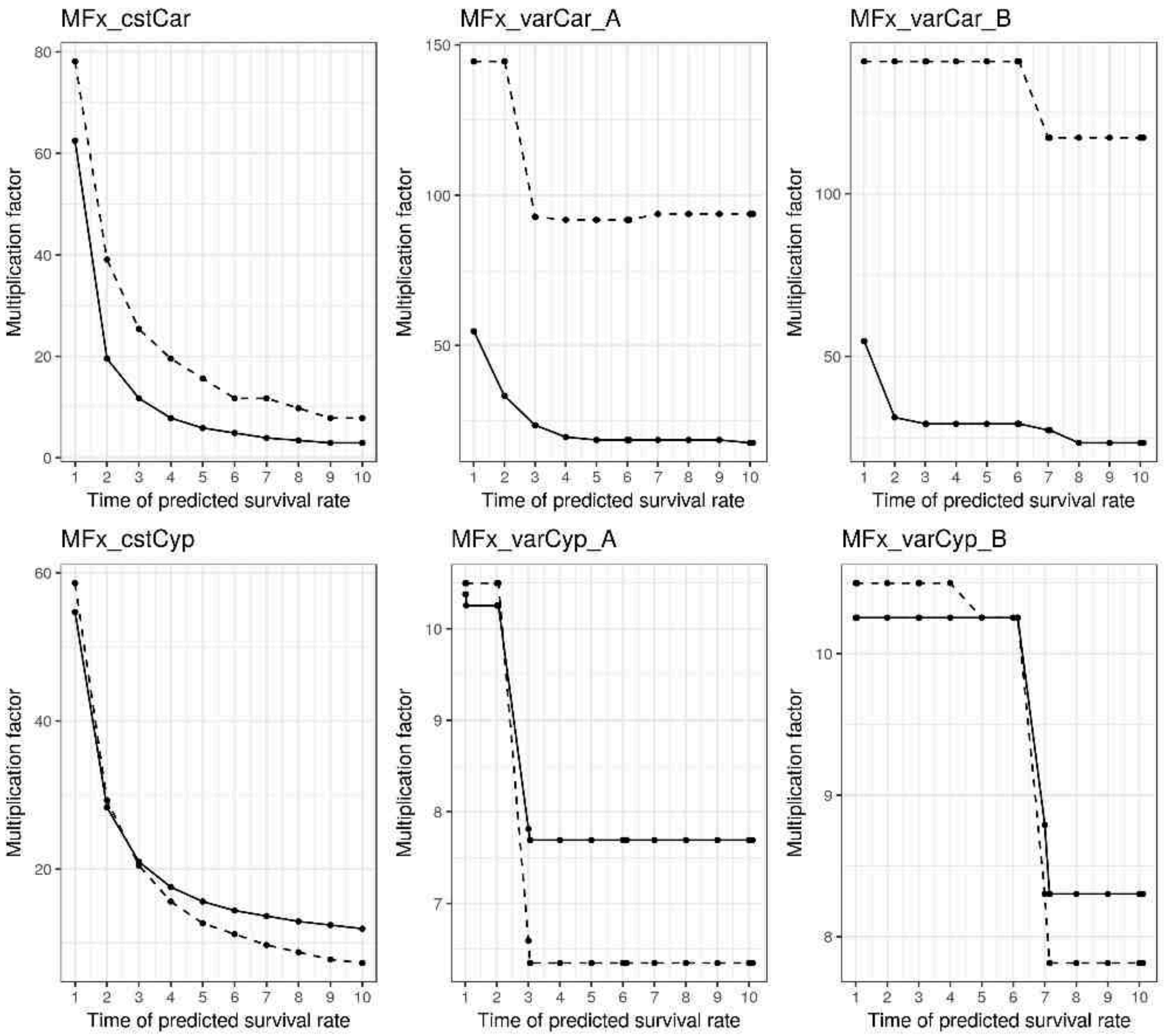

MFx_cstCar
MFx_varCar_A
MFx_varCar_B
MFx_cstCyp
MFx_varCyp_A
MFx_varCyp_B
Multiplication factor
Time of predicted survival rate

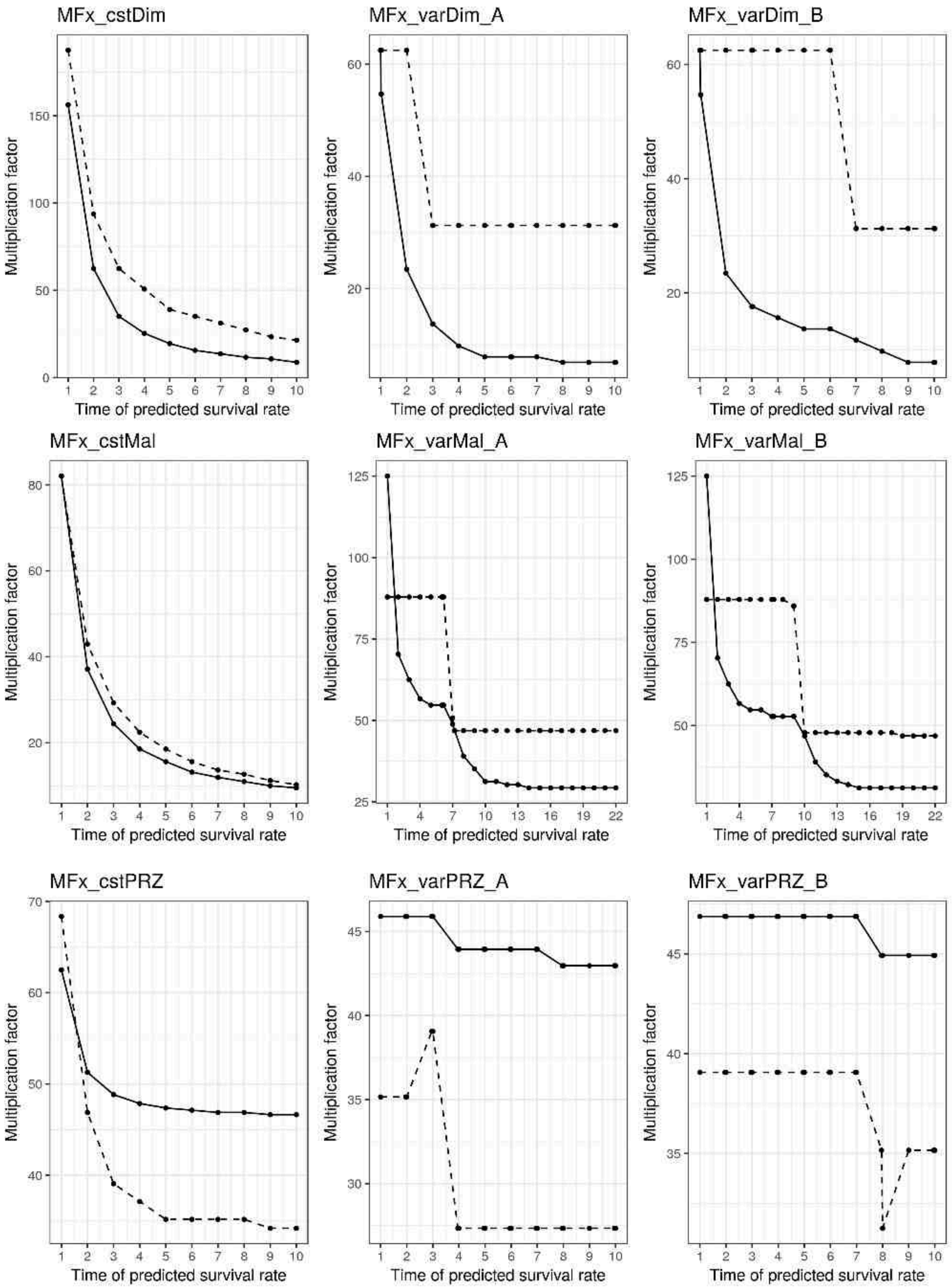

MFx_cstDim
MFx_varDim_A
MFx_varDim_B
MFx_cstMal
MFx_varMal_A
MFx_varMal_B
MFx_cstPRZ
MFx_varPRZ_A
MFx_varPRZ_B
Multiplication factor
Time of predicted survival rate

MFx along X

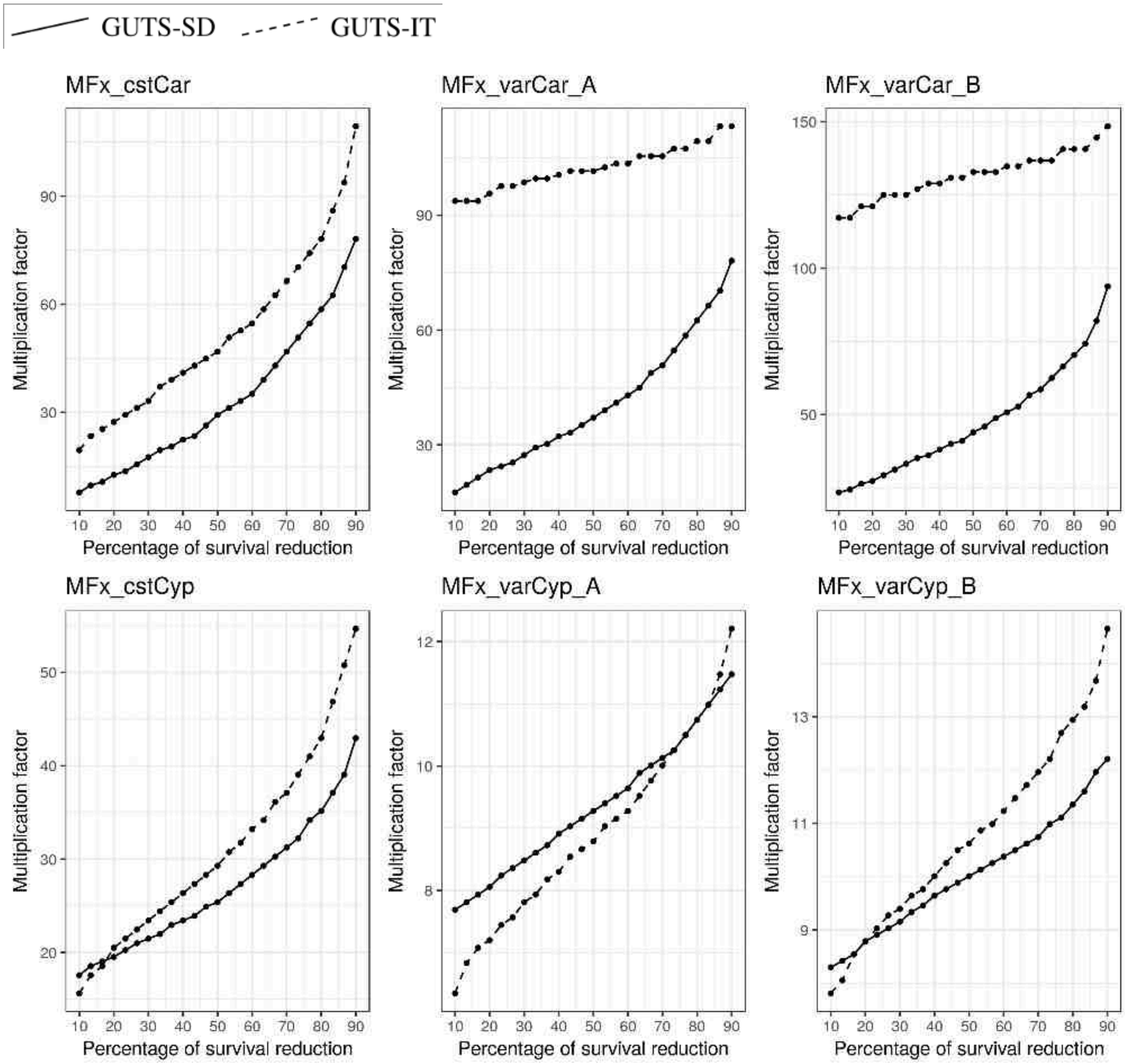
GUTS-SD
GUTS-IT
MFx_cstCar
MFx_varCar_A
MFx_varCar_B
MFx_cstCyp
MFx_varCyp_A
MFx_varCyp_B
Multiplication factor
Percentage of survival reduction

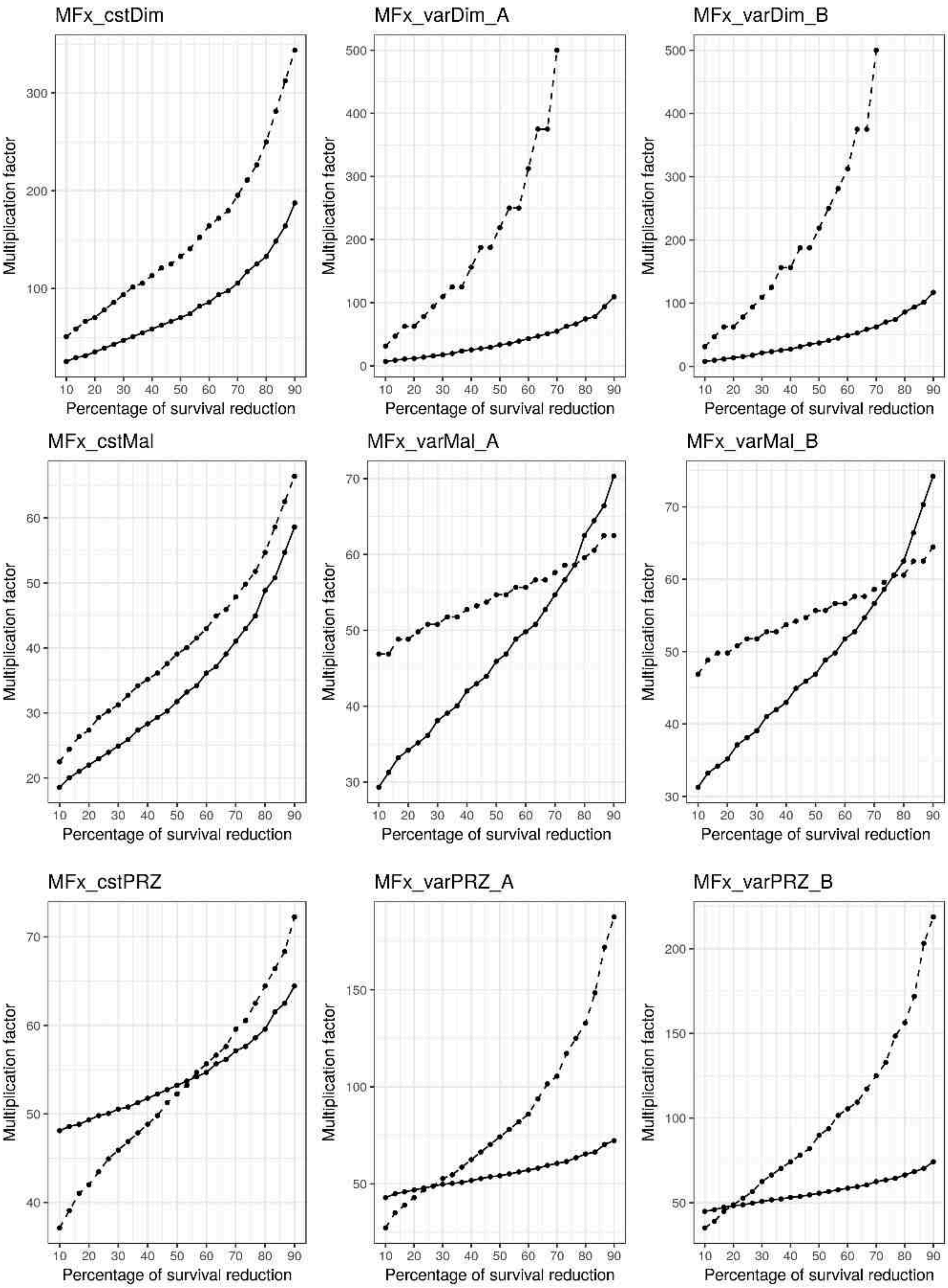
MFx_cstDim
MFx_varDim_A
MFx_varDim_B
MFx_cstMal
MFx_varMal_A
MFx_varMal_B
MFx_cstPRZ
MFx_varPRZ_A
MFx_varPRZ_B
Multiplication factor
Percentage of survival reduction

Predictions

Depuration times for every compounds

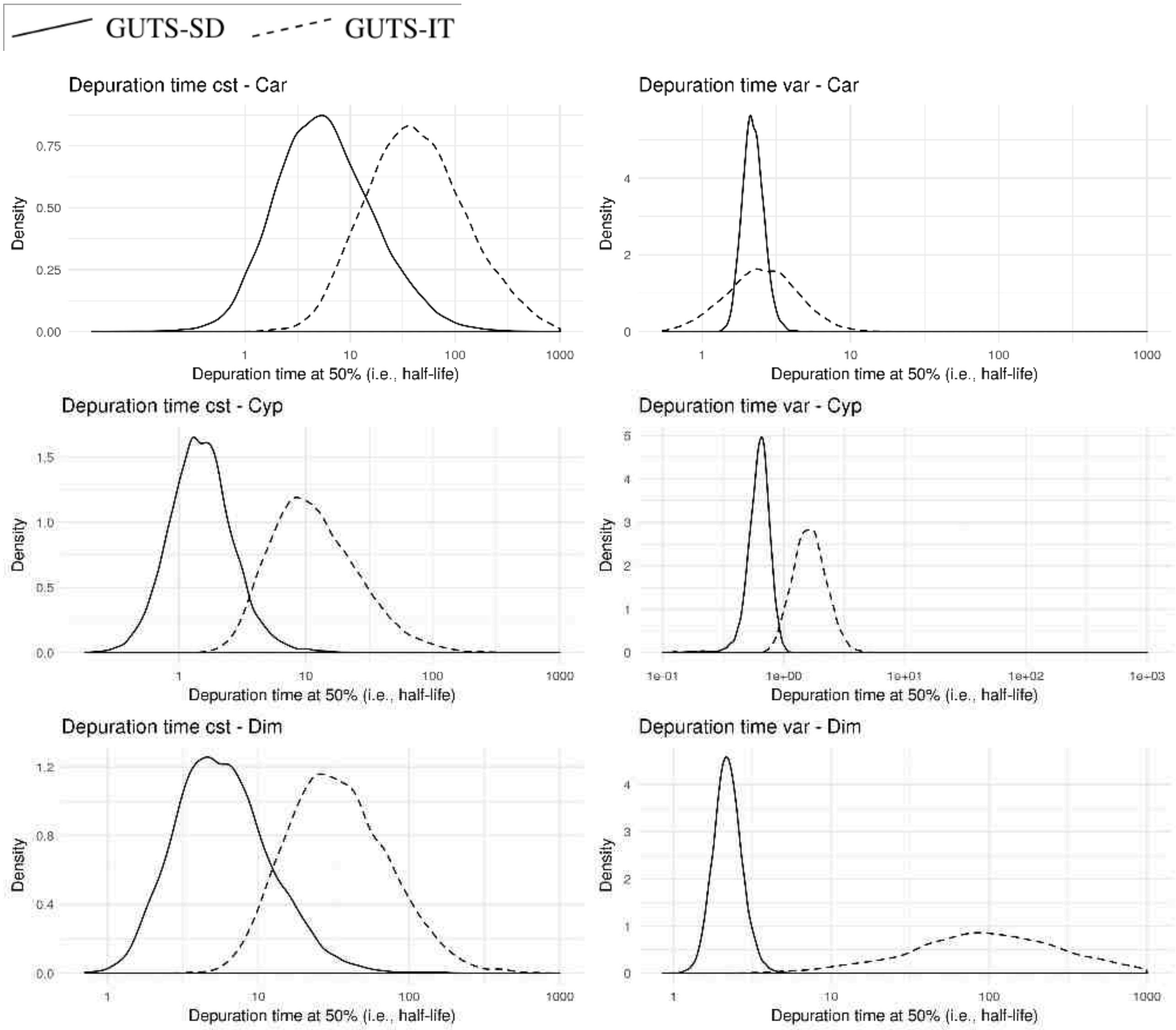

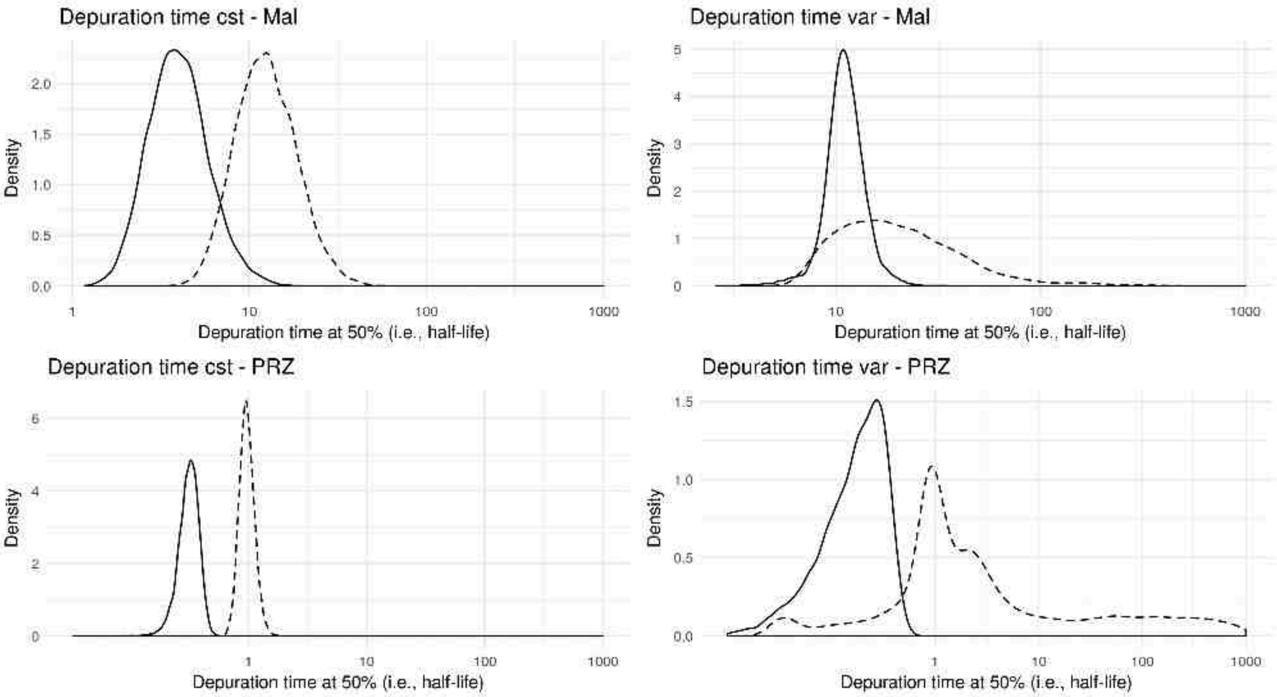

Prediction with different pulses frequencies and amplitudes

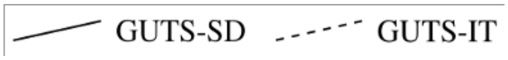

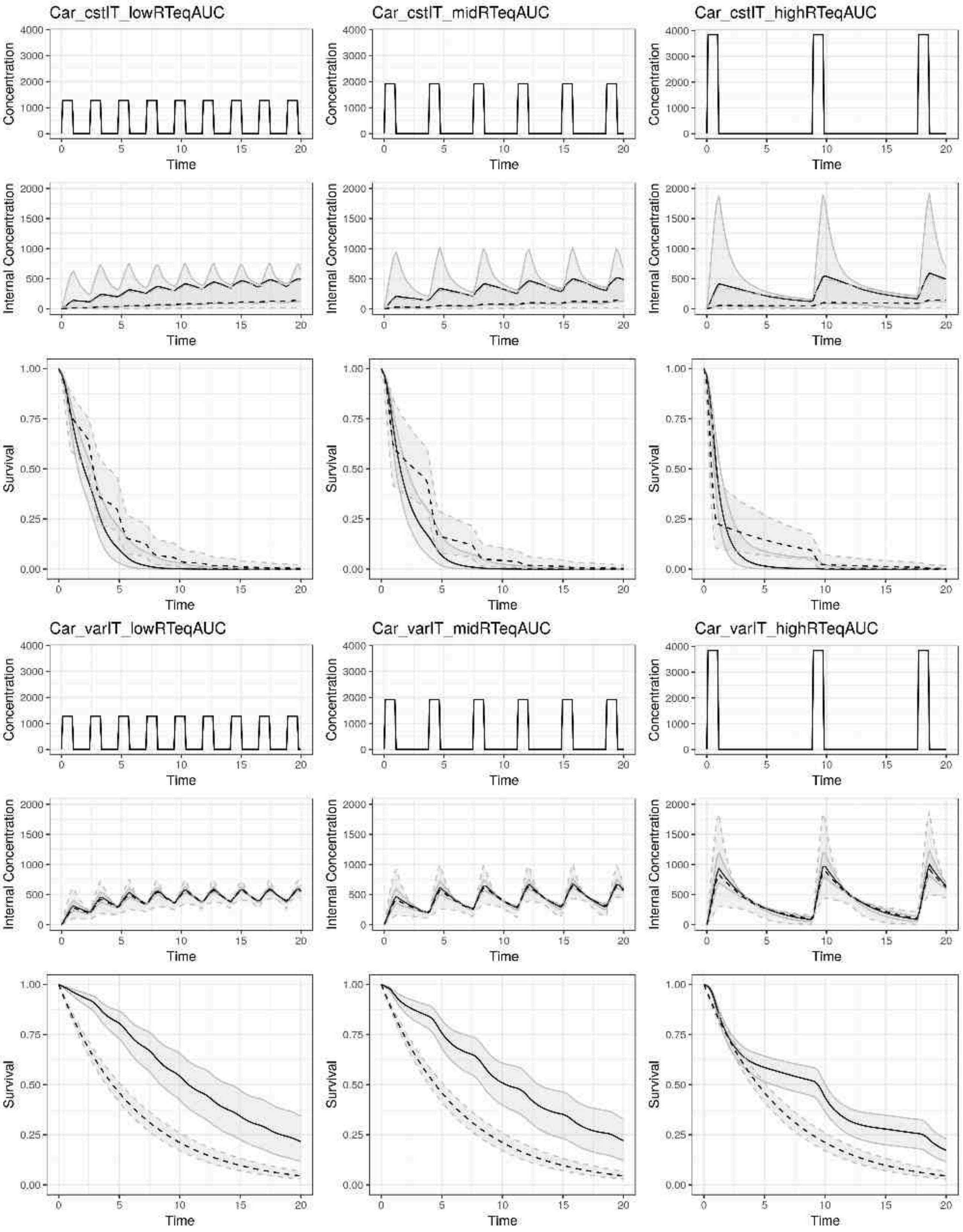

Car_cstIT_lowRTeqAUC
Car_cstIT_midRTeqAUC
Car_cstIT_highRTeqAUC
Car_varIT_lowRTeqAUC
Car_varIT_midRTeqAUC
Car_varIT_highRTeqAUC
Concentration
Internal Concentration
Survival
Time

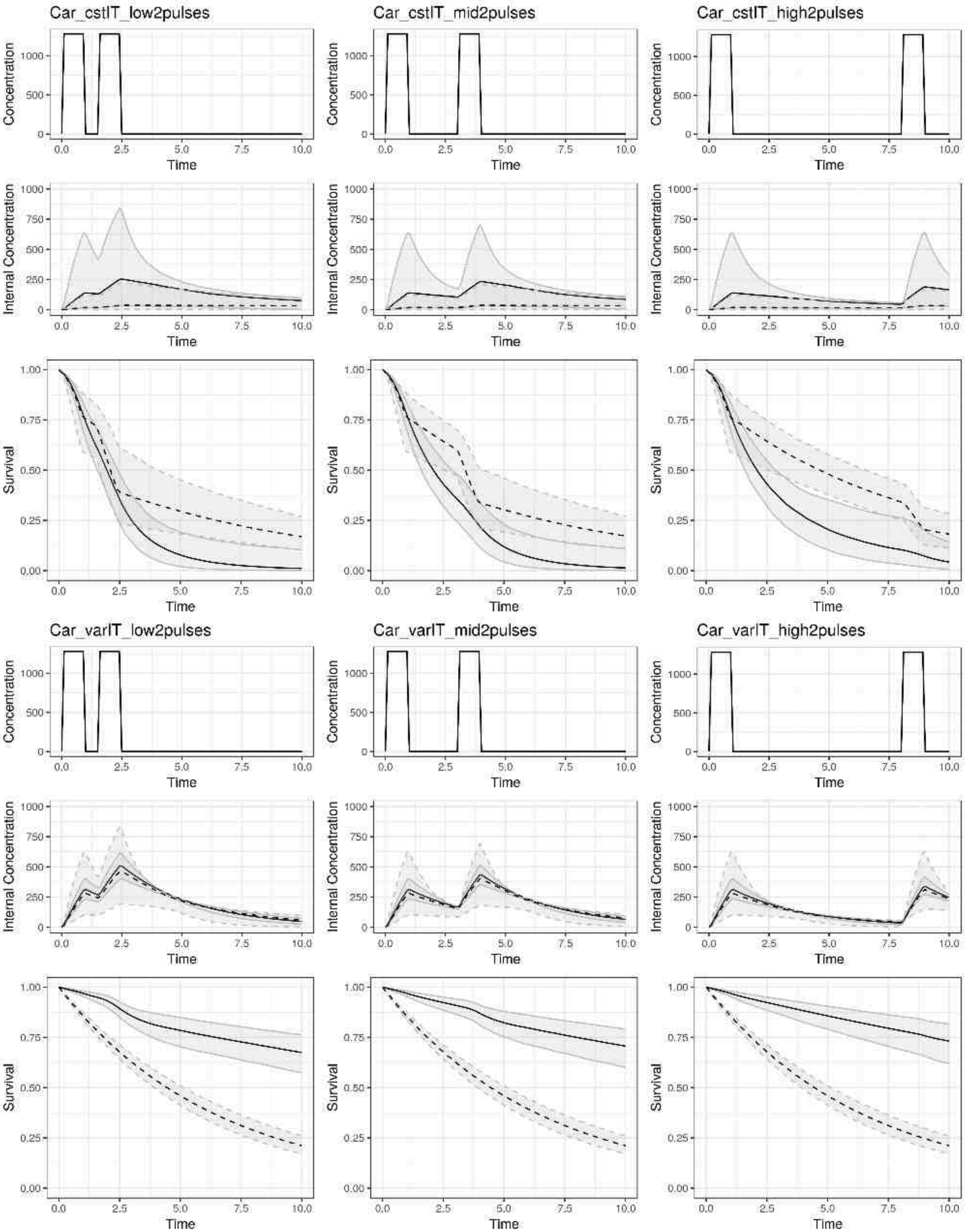

Car_cstIT_low2pulses
Car_cstIT_mid2pulses
Car_cstIT_high2pulses
Car_varIT_low2pulses
Car_varIT_mid2pulses
Car_varIT_high2pulses
Concentration
Internal Concentration
Survival
Time

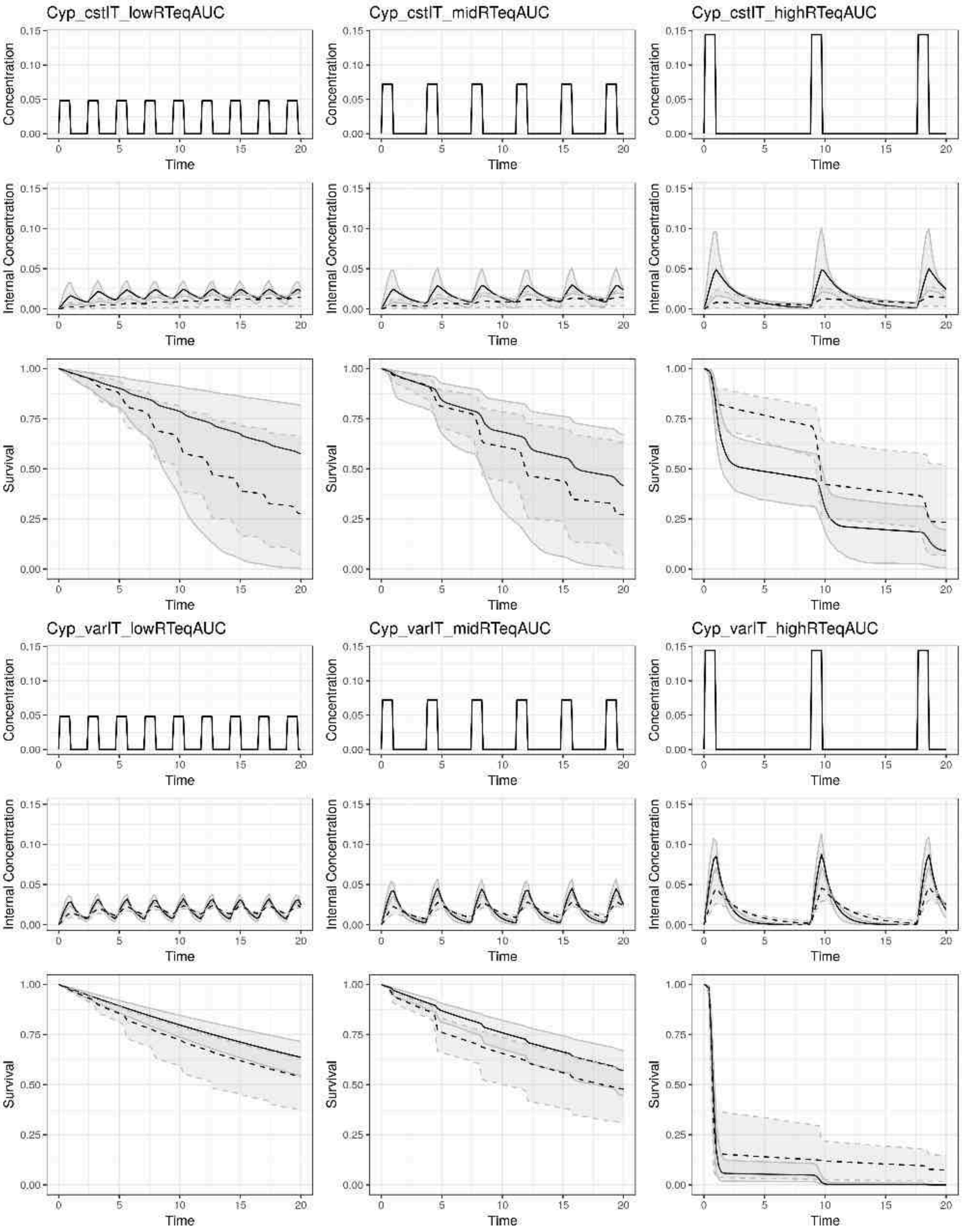
Cyp_cstIT_lowRTeqAUC
Cyp_cstIT_midRTeqAUC
Cyp_cstIT_highRTeqAUC
Cyp_varIT_lowRTeqAUC
Cyp_varIT_midRTeqAUC
Cyp_varIT_highRTeqAUC
Concentration
Internal Concentration
Survival
Time
0.00
0.05
0.10
0.15
0.25
0.50
0.75
1.00
0
5
10
15
20

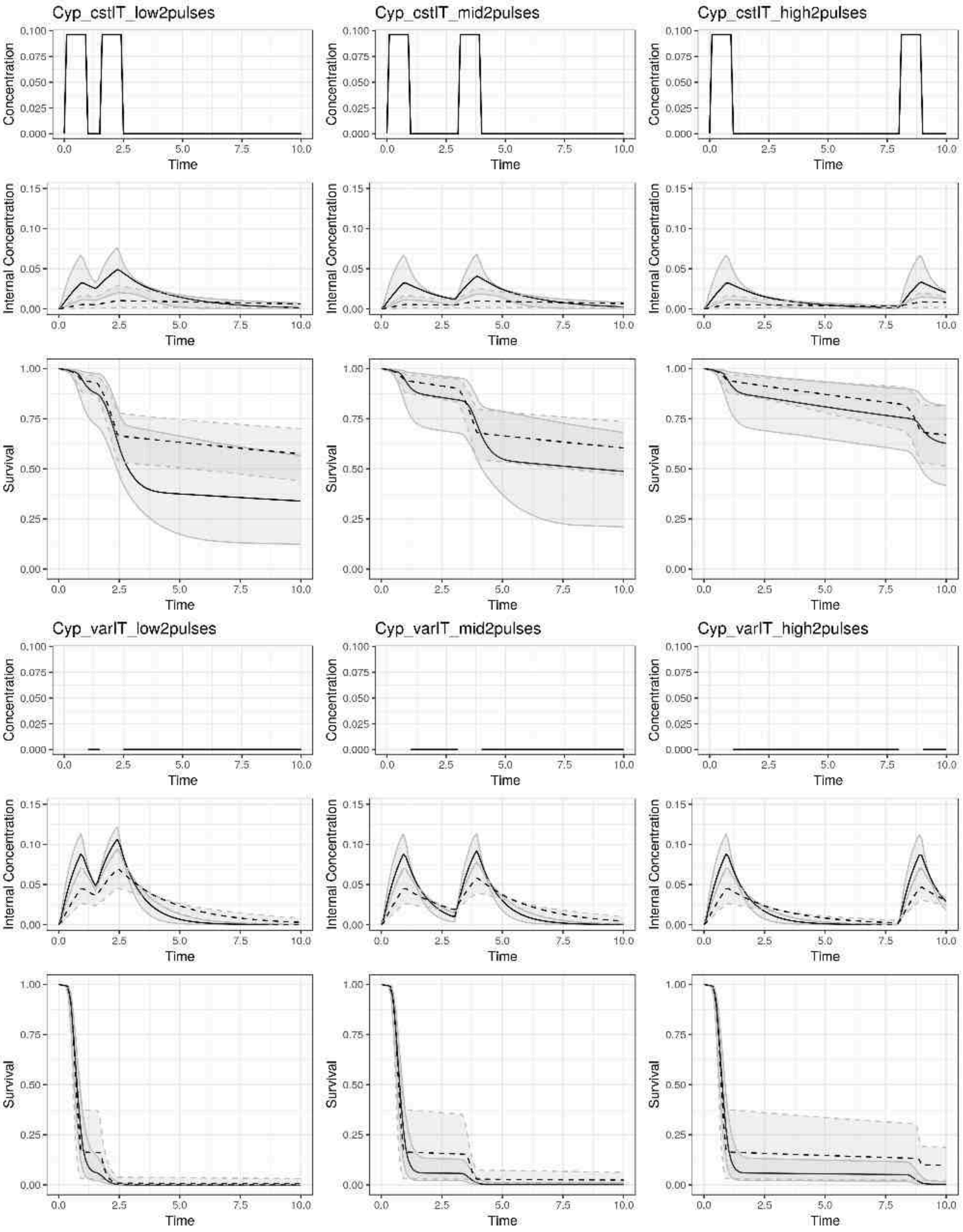

Cyp_cstIT_low2pulses
Cyp_cstIT_mid2pulses
Cyp_cstIT_high2pulses
Cyp_varIT_low2pulses
Cyp_varIT_mid2pulses
Cyp_varIT_high2pulses
Concentration
Internal Concentration
Survival
Time

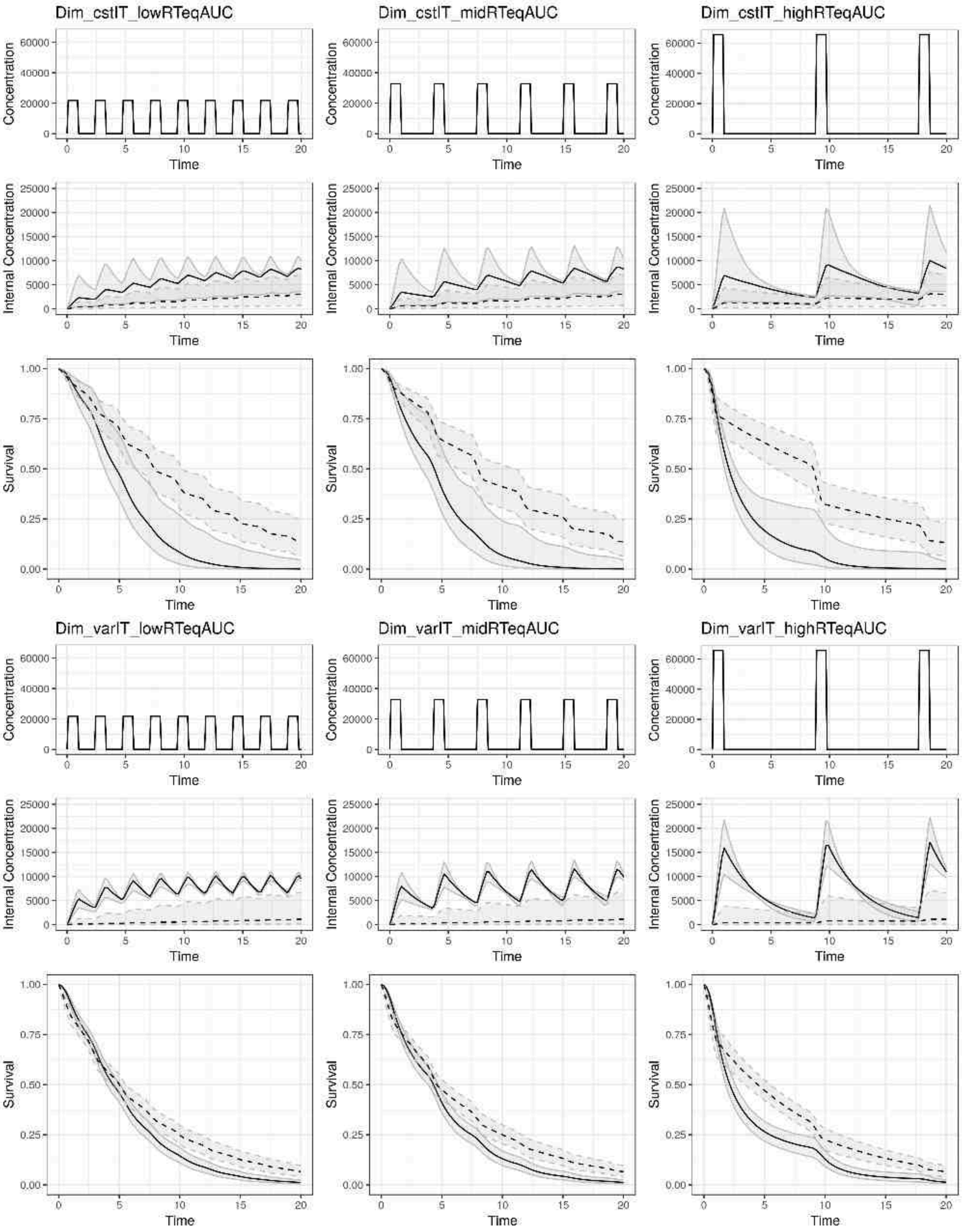

Dim_cstIT_lowRTeqAUC
Dim_cstIT_midRTeqAUC
Dim_cstIT_highRTeqAUC
Dim_varIT_lowRTeqAUC
Dim_varIT_midRTeqAUC
Dim_varIT_highRTeqAUC
Concentration
Internal Concentration
Survival
Time

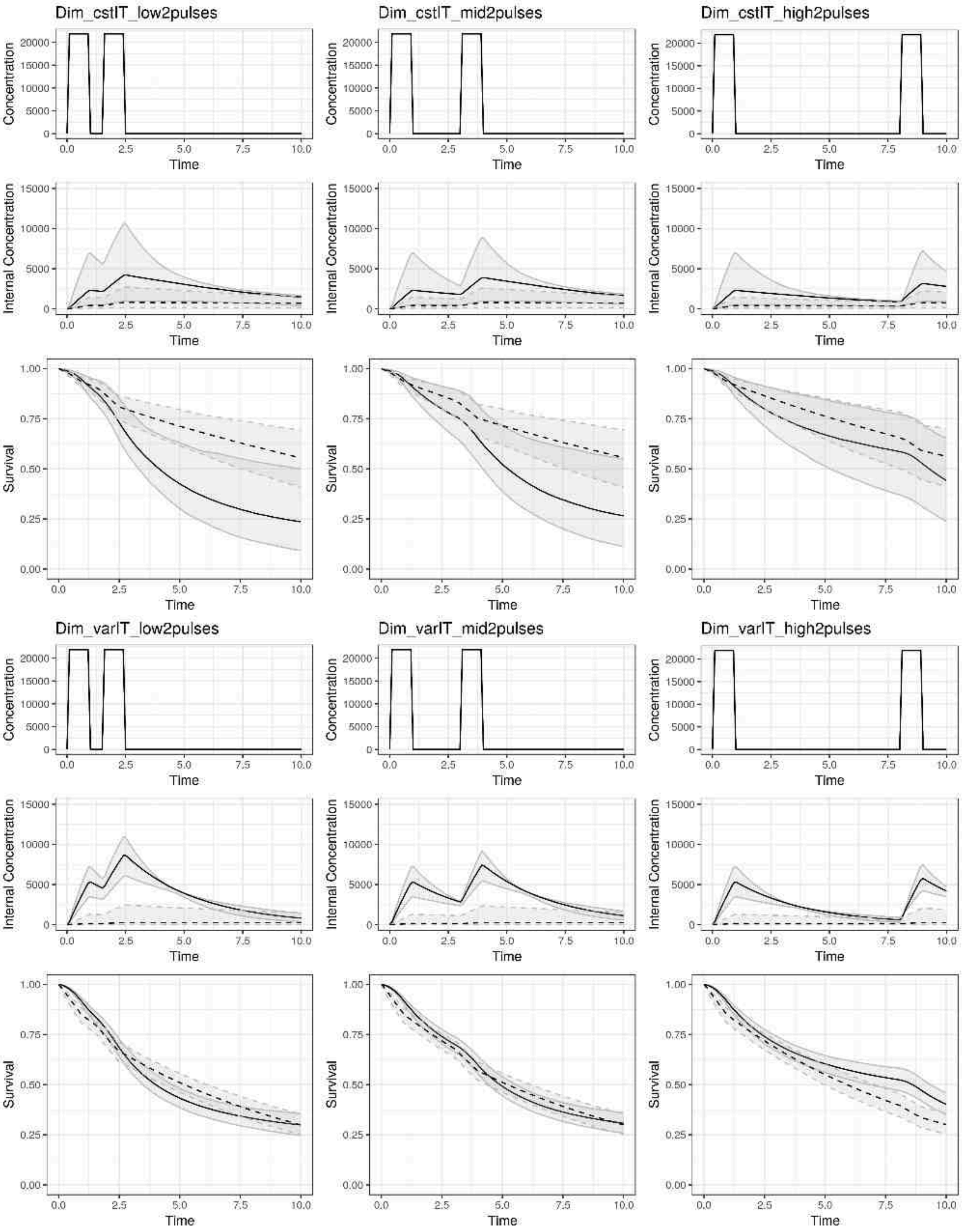
Dim_cstIT_low2pulses
Dim_cstIT_mid2pulses
Dim_cstIT_high2pulses
Dim_varIT_low2pulses
Dim_varIT_mid2pulses
Dim_varIT_high2pulses
Concentration
Internal Concentration
Survival
Time

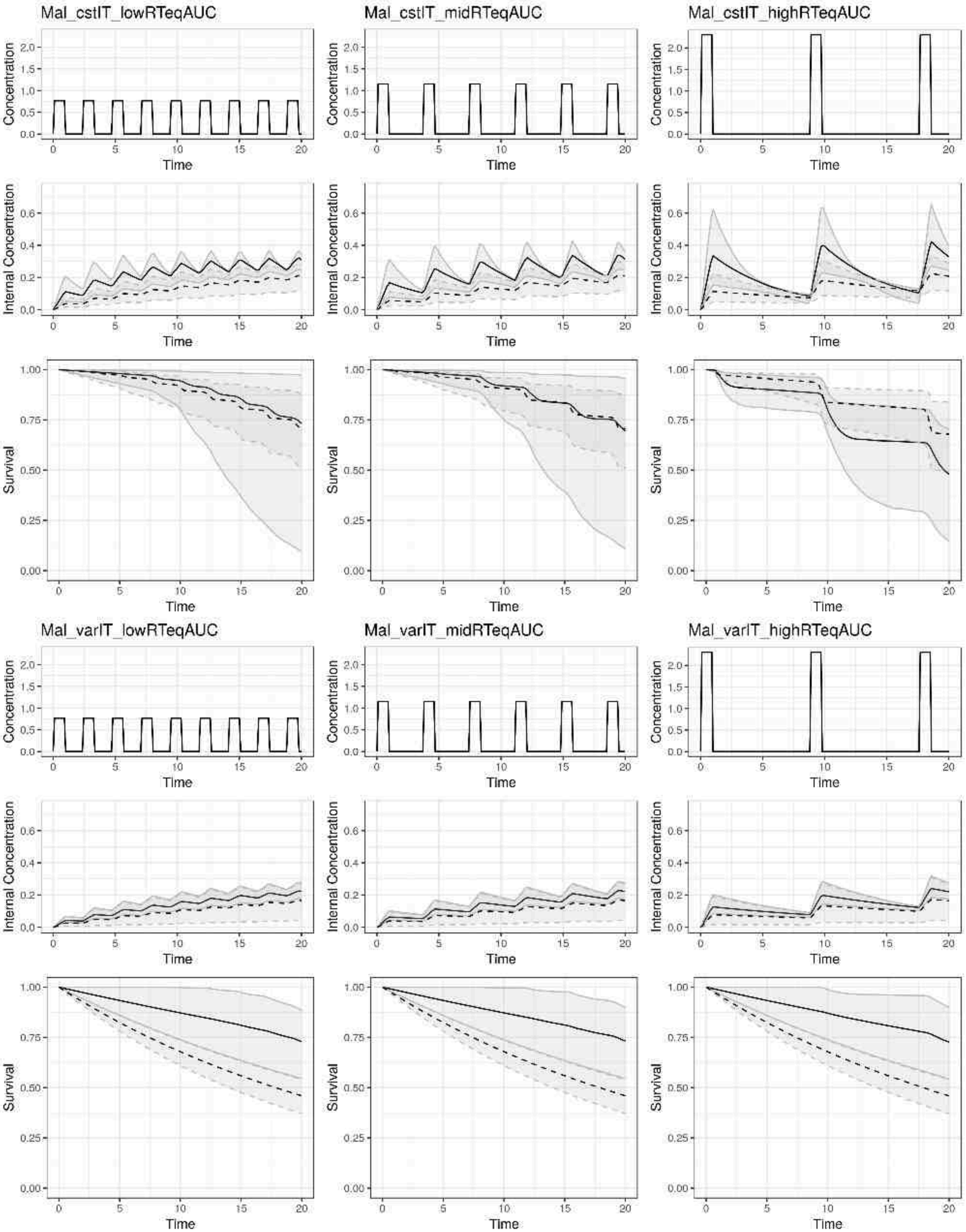

Mal_cstIT_lowRTeqAUC
Mal_cstIT_midRTeqAUC
Mal_cstIT_highRTeqAUC
Mal_varIT_lowRTeqAUC
Mal_varIT_midRTeqAUC
Mal_varIT_highRTeqAUC
Concentration
Internal Concentration
Survival
Time

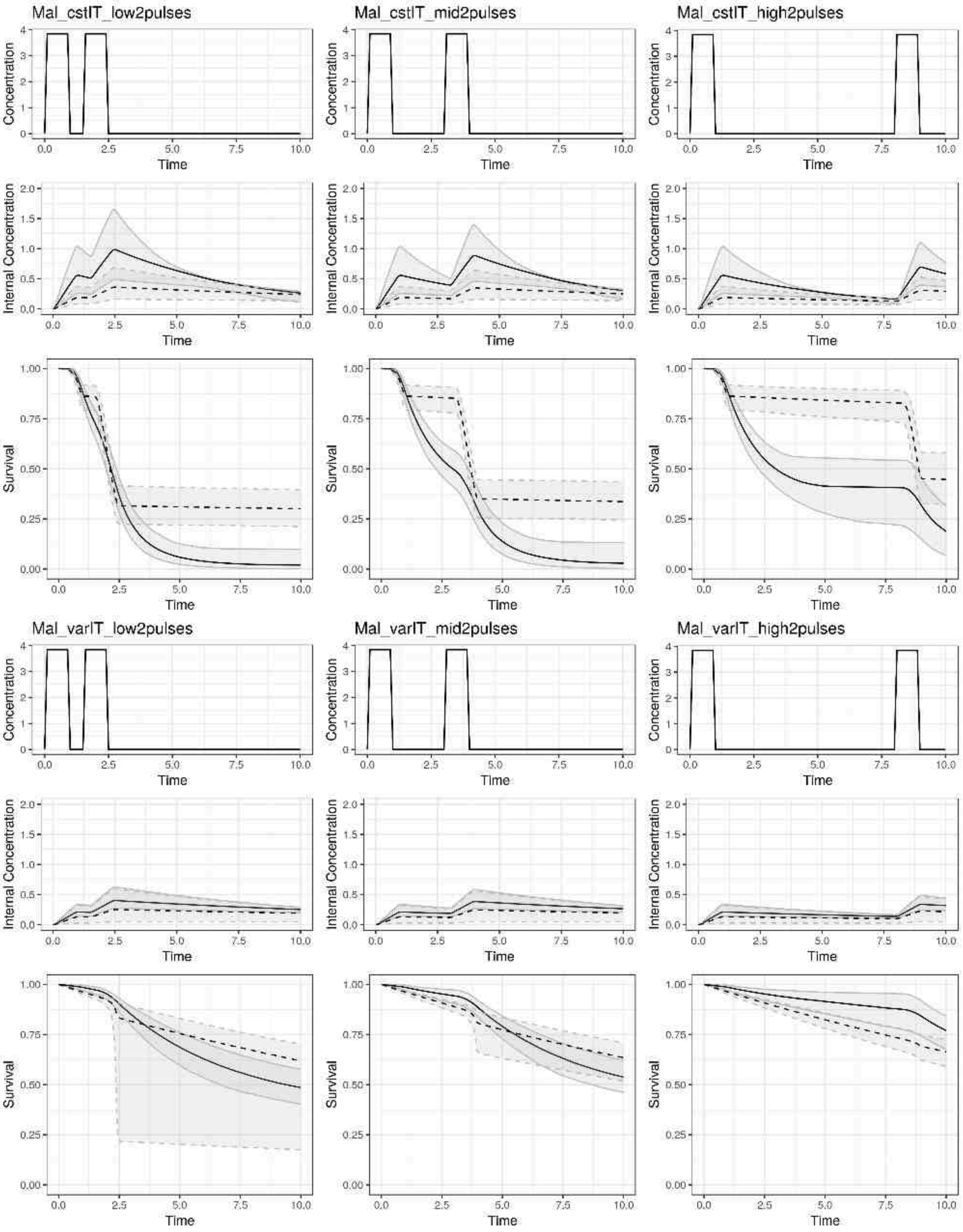
Mal_cstIT_low2pulses
Mal_cstIT_mid2pulses
Mal_cstIT_high2pulses
Mal_varIT_low2pulses
Mal_varIT_mid2pulses
Mal_varIT_high2pulses
Concentration
Internal Concentration
Survival
Time

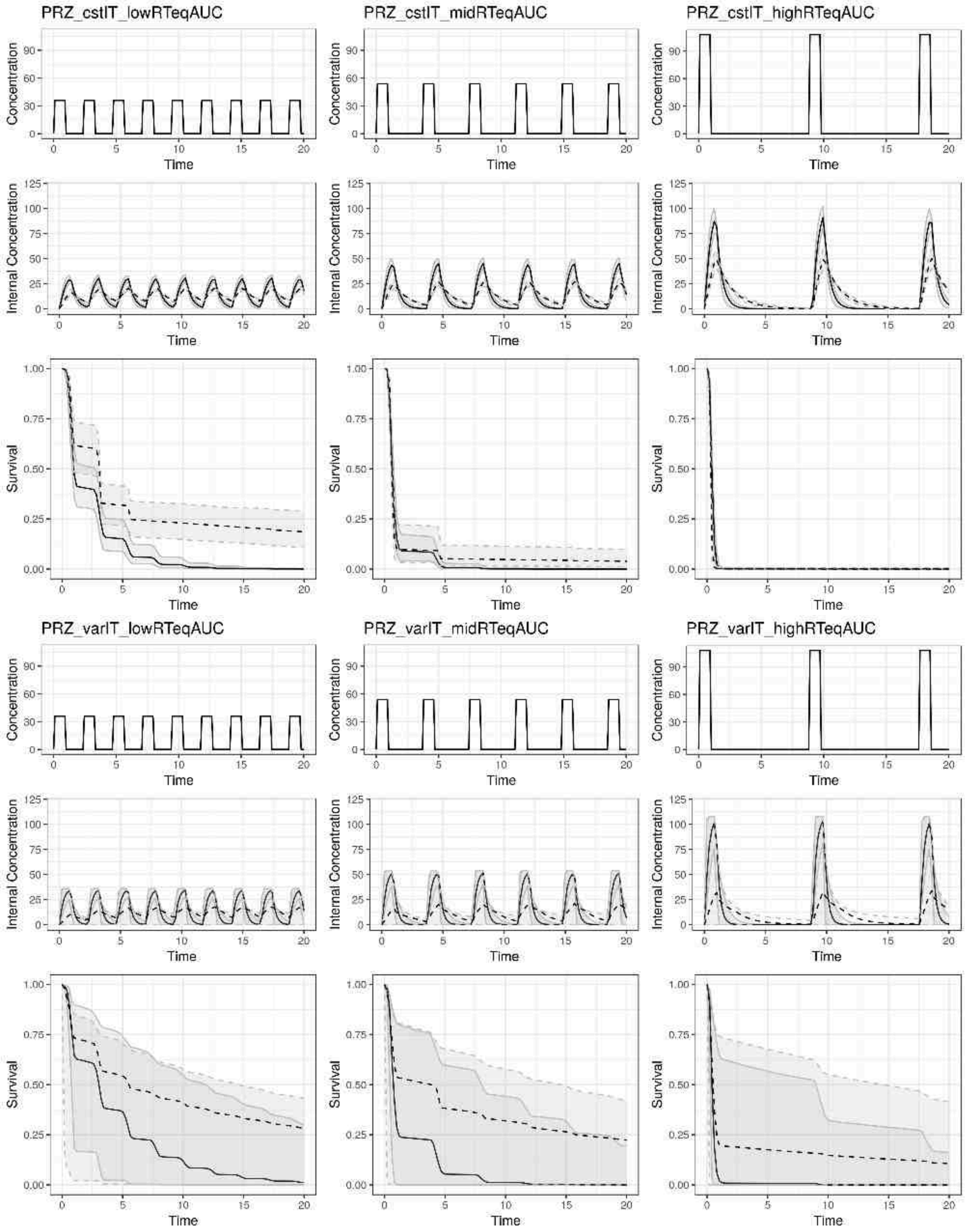
PRZ_cstIT_lowRTeqAUC
PRZ_cstIT_midRTeqAUC
PRZ_cstIT_highRTeqAUC
PRZ_varIT_lowRTeqAUC
PRZ_varIT_midRTeqAUC
PRZ_varIT_highRTeqAUC
Concentration
Internal Concentration
Survival
Time

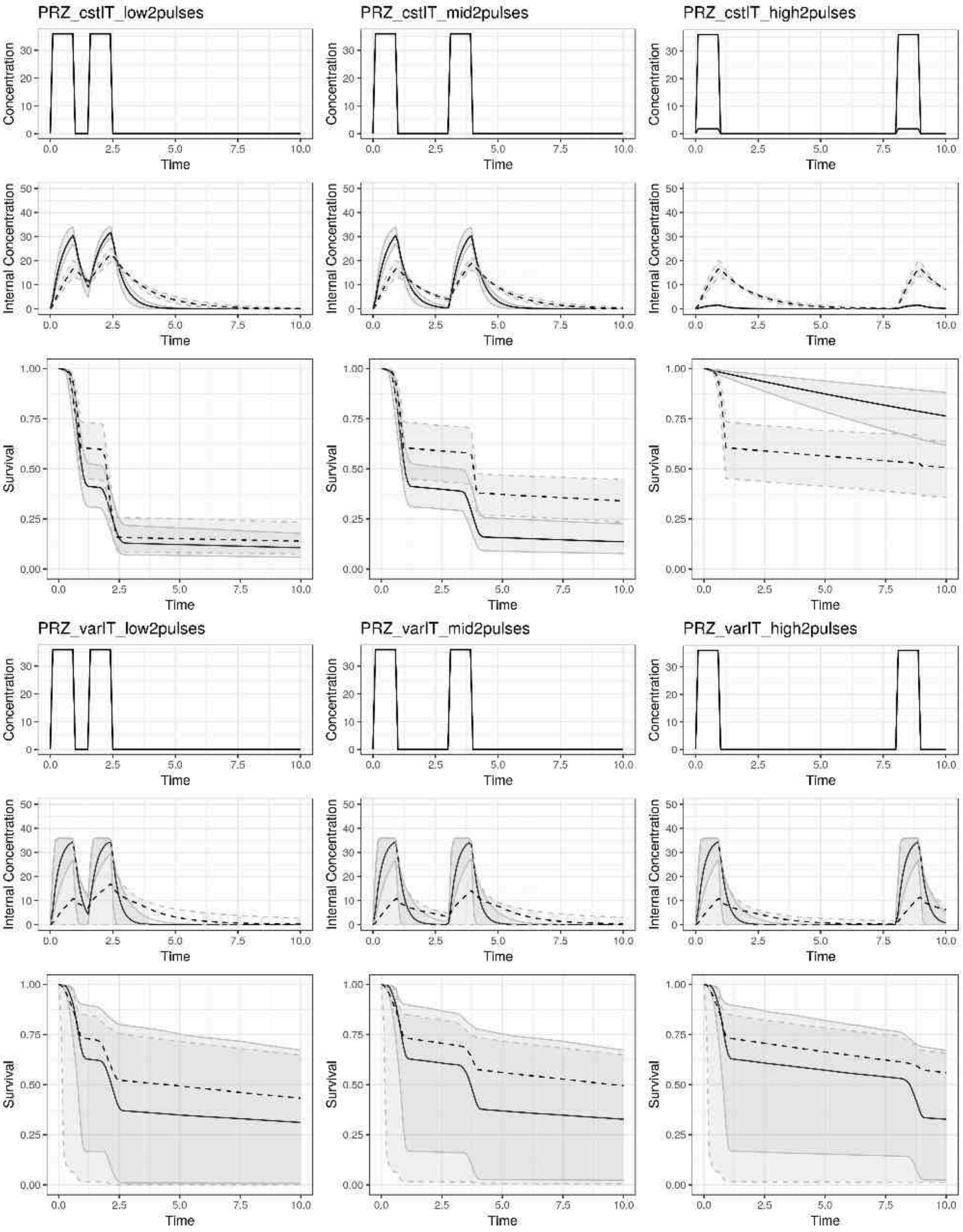
PRZ_cstIT_low2pulses
PRZ_cstIT_mid2pulses
PRZ_cstIT_high2pulses
PRZ_varIT_low2pulses
PRZ_varIT_mid2pulses
PRZ_varIT_high2pulses
Concentration
Internal Concentration
Survival
Time
