# Supplementary material for: Recommendations to address uncertainties in environmental risk assessment using toxicokinetic-toxicodynamic models
Source: Sci Rep. 2019 Aug 7;9:11432. doi: 10.1038/s41598-019-47698-0 (PMC6685980; doi:10.1038/s41598-019-47698-0)
Supplement: Supplementary file 2 — Supplementary Material - Mathematics [file 41598_2019_47698_MOESM2_ESM.pdf]

Supplementary Material - Mathematical analysis

Recommendations to address uncertainties in environmental risk assessment using toxicokinetic-toxicodynamic models

Virgile Baudrot[1,2,*] **and Sandrine Charles**[1]

[1]Univ Lyon, Université Lyon 1, UMR CNRS 5558, Laboratoire de Biométrie et Biologie Évolutive, F-69100 Villeurbanne, France

[2] INRA, BioSP, 84000 Avignon, France

Corresponding author 1 Virgile Baudrot
Mail: virgile.baudrot@posteo.net

Corresponding author 2 Sandrine Charles
Mail: sandrine.charles@univ-lyon1.fr

1 Mathematical notations in reduced GUTS models

In this Supplementary Material concerning the derivation of mathematical equations, we use the following notations:

Table S1: Mathematical notations and descriptions

Notation	Description
h_b	Background hazard rate (intrinsic mortality rate)
k_d	Dominant rate constant for the reduced GUTS model
b_w	Killing rate, referenced to external concentration
z	Threshold for effect
m_w	Median of the distribution of threshold
β	Shape parameter for the distribution threshold
C_w	External chemical concentration in the environment
D_w	Scaled damage, referenced to external concentration
$S(C_w, t)$	Survival probability in a population of individuals ; $S_{SD}()$, $S_{IT}()$ with models $GUTS - RED - SD$, $GUTS - RED - IT$, reps.
$LC(x, t)$	Lethal Concentration for $x\%$ of organisms at time t ; $LC_{SD}()$, $LC_{IT}()$ with models $GUTS - RED - SD$, $GUTS - RED - IT$, reps.
$MF(x, t)$	Multiplication Factor leading to an increase of $x\%$ of additional mortality at time t ; $MF_{SD}()$, $MF_{IT}()$ with models $GUTS - RED - SD$, $GUTS - RED - IT$, reps.

The dynamic of the internal damage (i.e. the toxicokinetic) is given by:

$$\frac{dD_w(t)}{dt} = k_d \left(C_w(t) - D_w(t) \right) \tag{S1}$$

We also suppose that $D_w(0) = 0$. Therefore, when the external concentration is constant (i.e. $C_w(t) = C_w$, we have:

$$D_w(t) = C_w \left(1 - e^{-k_d t} \right) \tag{S2}$$

Otherwise, if the external concentration is varying with time, we have:

$$D_w(t) = e^{-k_d t} k_d \int_0^t e^{k_d \tau} C_w(\tau)\, d\tau \tag{S3}$$

2 Mathematical analysis of $LC(x, t)$

The lethal concentration makes sense only in the case of constant exposure profile (i.e. $C_w(t) = C_w$, $\forall\, t$).

Definition 1 ($LC(x, t)$)**.** *The lethal concentration for $x\%$ of organisms at time t is the function denoted $LC(x, t)$ and defined (using notations provided in Table S1) as:*

$$S(LC(x, t), t) = S(0, t) \left(1 - \frac{x}{100} \right) \tag{S4}$$

Where $S(LC(x,t),t)$ is the survival rate at time t when the internal concentration is continuously the constant $LC(x,t)$ from time 0 to time t. Also, $S(0,t)$ is the survival rate at time t when there is no contaminant (i.e. external concentration equals 0 all along the experiment), which is defined from the background mortality rate:

$$S(0,t) = \exp\left(-\int_0^t h_b d\tau\right) = e^{-h_b t} \tag{S5}$$

2.1 $LC_{SD}(x,t)$ for model GUTS-RED-SD

For GUTS-RED-SD model (with $S_{SD}(C,t)$ the survival rate for GUTS-RED-SD), the $LC_{SD}(x,t)$ is given by:

$$\begin{aligned} & S_{SD}(LC_{SD}(x,t),t) = S_{SD}(0,t)\left(1-\frac{x}{100}\right) \\ \Leftrightarrow \quad & \exp\left(-\int_0^t b_w \max_{0<\tau<t}(D_w(\tau)-z,0)d\tau\right) e^{-h_b t} = e^{-h_b t}\left(1-\frac{x}{100}\right) \\ \Leftrightarrow \quad & \int_0^t \max_{0<\tau<t}(D_w(\tau)-z,0)\, d\tau = \frac{-1}{b_w}\ln\left(1-\frac{x}{100}\right) \end{aligned} \tag{S6}$$

Then, with t_z, the time at which the internal concentration is equal to the threshold, $D_w(t_z) = z$, using (S1), we have:

$$t_z = \frac{-1}{k_d}\ln\left(1-\frac{z}{LC_{SD}(x,t)}\right) \tag{S7}$$

And equation (S6) can be developped as:

$$\begin{aligned} \text{(S6)} \Leftrightarrow \quad & \int_{t_z}^t \left(LC_{SD}(x,t)\left(1-e^{-k_d\tau}\right)-z\right)\, d\tau = \frac{-1}{b_w}\ln\left(1-\frac{x}{100}\right) \\ \Leftrightarrow \quad & LC_{SD}(x,t)(t-t_z) - z(t-t_z) + \frac{LC_{SD}(x,t)}{k_d}\left(e^{-k_d t}-e^{-k_d t_z}\right) = \frac{-1}{b_w}\ln\left(1-\frac{x}{100}\right) \\ \Leftrightarrow \quad & LC_{SD}(x,t)\left(t-t_z+\frac{1}{k_d}\left(e^{-k_d t}-e^{-k_d t_z}\right)\right) - z(t-t_z) = \frac{-1}{b_w}\ln\left(1-\frac{x}{100}\right) \\ \Leftrightarrow \quad & LC_{SD}(x,t) = \frac{-k_d\ln\left(1-\frac{x}{100}\right)}{b_w\left(k_d(t-t_z)-e^{-k_d t_z}+e^{-k_d t}\right)} + \frac{k_d z(t-t_z)}{k_d(t-t_z)-e^{-k_d t_z}+e^{-k_d t}} \end{aligned} \tag{S8}$$

The expression of t_z prevents to have an explicit formulation of $LC_{SD}(x,t)$.

We can use equation (S7) to see that:

$$e^{-k_d t_z} = \exp\left(-k_d\frac{-1}{k_d}\ln\left(1-\frac{z}{LC_{SD}(x,t)}\right)\right) = 1-\frac{z}{LC_{SD}(x,t)} \tag{S9}$$

Combining this result with equation (S8), we obtain:

$$LC_{SD}(x,t) = \frac{\frac{-k_d}{b_w}\ln\left(1-\frac{x}{100}\right)+z(k_d(t-t_z)-1)}{k_d(t-t_z)+e^{-k_d t}-1} \tag{S10}$$

2.1.1 Convergence of $LC_{SD}(x,t)$ when t tends to infinity

We assume the threshold concentration, z, is reached in a finite time, which mean that $\lim_{t\to+\infty} t-t_z = +\infty$.

To compute $\lim_{t \to +\infty} LC_{SD}(x,t)$, we have for one part of the equation:

$$\lim_{t \to +\infty} \frac{\dfrac{-k_d}{b_w} \ln\left(1 - \dfrac{x}{100}\right)}{k_d(t - t_z) + e^{-k_d t} - 1} = 0 \tag{S11}$$

And for the other part of the equation, we have:

$$\lim_{t \to +\infty} \frac{z(k_d(t - t_z) - 1)}{k_d(t - t_z) + e^{-k_d t} - 1} = \lim_{t \to +\infty} \frac{z}{\dfrac{e^{-k_d t}}{k_d(t - t_z) - 1} + 1} = z \tag{S12}$$

As a consequence, we obtain:

$$\lim_{t \to +\infty} LC_{SD}(x,t) = z \tag{S13}$$

2.1.2 Influence of x on the $LC_{SD}(x,t)$

Sensitivity:

To study the influence of x on the $LC_{SD}(x,t)$, we compute the sensitivity, which is simply the derivative of $LC_{SD}(x,t)$ by x.

$$\frac{\partial LC_{SD}(x,t)}{\partial x} = \frac{k_d}{(100 - x) b_w \left(k_d(t - t_z) - e^{-k_d t_z} + e^{-k_d t}\right)} \tag{S14}$$

Since $t \geq t_z$, then $b_w k_d(t - t_z) \geq 0$ while $e^{-k_d t} - e^{-k_d t_z} \leq 0$, and therefore the sign of the derivative may change according to the parameter values.

Elasticity:

The elasticity of LC_{SD} to parameter x represents the proportional change in LC_{SD} in response to a proportional change in the parameter x and is given by:

$$\frac{\partial LC_{SD}(x,t)}{\partial x} \times \frac{x}{LC_{SD}(x,t)} = \frac{x}{(100 - x)\left(b_w z(t - t_z) - \ln\left(1 - \frac{x}{100}\right)\right)} \tag{S15}$$

2.2 $LC_{IT}(x,t)$ for model GUTS-RED-IT

For GUTS-RED-IT model (with $S_{IT}(C,t)$ the survival rate for GUTS-RED-IT), the $LC_{IT}(x,t)$ is given by:

$$
\begin{aligned}
& & S_{IT}(LC_{IT}(x,t),t) &= S_{IT}(0,t)\left(1-\frac{x}{100}\right) \\
&\Leftrightarrow & e^{-h_b t}\left(1-\frac{1}{1+\left(\frac{LC_{IT}(x,t)(1-e^{-k_d t})}{m_w}\right)^{-\beta}}\right) &= e^{-h_b t}\left(1-\frac{x}{100}\right) \\
&\Leftrightarrow & 1-\frac{1}{1+\left(\frac{LC_{IT}(x,t)(1-e^{-k_d t})}{m_w}\right)^{-\beta}} &= 1-\frac{x}{100} \\
&\Leftrightarrow & 1+\left(\frac{LC_{IT}(x,t)(1-e^{-k_d t})}{m_w}\right)^{-\beta} &= \frac{100}{x} \\
&\Leftrightarrow & \left(\frac{LC_{IT}(x,t)(1-e^{-k_d t})}{m_w}\right)^{-\beta} &= \frac{100-x}{x} \\
&\Leftrightarrow & \left(\frac{LC_{IT}(x,t)(1-e^{-k_d t})}{m_w}\right)^{\beta} &= \frac{x}{100-x} \\
&\Leftrightarrow & \frac{LC_{IT}(x,t)(1-e^{-k_d t})}{m_w} &= \sqrt[\beta]{\frac{x}{100-x}} \\
&\Leftrightarrow & LC_{IT}(x,t) &= \frac{m_w}{(1-e^{-k_d t})}\sqrt[\beta]{\frac{x}{100-x}}
\end{aligned} \tag{S16}
$$

2.2.1 Convergence of $LC_{IT}(x,t)$ when t tends to infinity

Using equation (S16), we can direclty see that

$$\lim_{t\to+\infty} LC_{IT}(x,t) = m_w \sqrt[\beta]{\frac{x}{100-x}} \tag{S17}$$

For the specific case of $x = 50\%$, we have:

$$\lim_{t\to+\infty} LC_{IT}(50,t) = m_w \tag{S18}$$

2.2.2 Influence of x on $LC_{IT}(x,t)$

Sensitivity:

As for the $LC_{SD}(x,t)$, we compute the sensitivity of $LC_{IT}(x,t)$ for parameter x by mean of the derivative:

$$\frac{\partial LC_{IT}(x,t)}{\partial x} = \frac{-100 m_w e^{k_d t}\sqrt[\beta]{\frac{x}{100-x}}}{\beta(100-x)x(1-e^{k_d t})} \tag{S19}$$

Elasticity:

Here, for this model, we can simplify the equation of sensitivity by computing the elasticity which is given by:

$$\frac{\partial LC_{IT}(x,t)}{\partial x} \times \frac{x}{LC_{IT}(x,t)} = \frac{-100 e^{k_d t}(1-e^{-k_d t})}{\beta(100-x)(1-e^{k_d t})} = \frac{100}{\beta(100-x)} \tag{S20}$$

The elasticity of $LC_{IT}(x,t)$ to parameter x represents the proportional change of $LC_{IT}(x,t)$ in response to a proportional change in x. Here, the elasticity of $LC_{IT}(x,t)$ to x is only function of β and x.

3 Mathematical analysis of $MF(x,t)$

Contrary to the lethal concentration $LC(x,t)$, the multiplication factor, denoted $MF(x,t)$ makes sense for any type of exposure profile (constant or time-variable).

Definition 2 ($MF(x,t)$)**.** *The multiplication factor leading to a reduction of $x\%$ of the survival rate at time t is the function denoted $MF(x,t)$ and defined (using notations provided in Table S1) as:*

$$S\left(MF(x,t)\times C_w(\tau),t\right)=S\left(C_w(\tau),t\right)\left(1-\frac{x}{100}\right) \tag{S21}$$

where $C_w(\tau)$ is the exposure profile along the continuous period from 0 to t. The expression $S(MF(x,t)\times C_w(\tau),t)$ is the survival rate at time t when the internal concentration is the exposure profile $C_w(\tau)$ multiplied by the constant multiplication factor $MF(x,t)$.

3.1 Internal concentration scaled by Multiplication Factor

The internal damage (or scaled internal concentration) at time t, $D_w(t)$, is given by equation (S1) using the profile of external concentration, $C_w(\tau)$, with τ going from 0 to t.

If the profile of external concentration is multiplied by a factor $MF(x,t)$ (assuming it reduces the survival rate by $x\%$ at time t), then, the internal concentration, denoted $D_w^{MF}(t)$, is also multiplied by this factor. From equation (S1) we get:

$$\frac{d\,D_w^{MF}(t)}{d\,t}=k_d(MF(x,t)\times C_w(\tau)-D_w^{MF}(t)) \quad \Rightarrow \quad D_w^{MF}(t)=MF(x,t)\times e^{-k_d t}\int_0^t k_d e^{k_d\tau}C_w(\tau)d\tau \tag{S22}$$

what leads to:

$$D_w^{MF}(t)=MF(x,t)\times D_w(t) \tag{S23}$$

Therefore, the internal damage referenced to the external concentration $D_w(t)$ is linearly scaled by the multiplication factor $MF(x,t)$ whatever the exposure profile $C_w(\tau)$.

3.2 $MF_{SD}(x,t)$ for model GUTS-RED-SD

Using the definition of the multiplication factor, we have the following formulation for the GUTS-RED-SD model:

$$\begin{aligned}
& S_{SD}\left(MF_{SD}(x,t)\times C_w(\tau),t\right)=S_{SD}(C_w(\tau),t)\left(1-\frac{x}{100}\right)\\
\Leftrightarrow\quad & \exp\left(-\int_0^t b_w \max_{0<\tau<t}(MF_{SD}(x,t)D_w(\tau)-z\,,\,0)+h_b\,d\tau\right)=\exp\left(-\int_0^t b_w \max_{0<\tau<t}(D_w(\tau)-z\,,\,0)+h_b\,d\tau\right)\left(1-\frac{x}{100}\right)\\
\Leftrightarrow\quad & \int_0^t b_w \max_{0<\tau<t}(MF_{SD}(x,t)D_w(\tau)-z\,,\,0)\,d\tau=\int_0^t b_w \max_{0<\tau<t}(D_w(\tau)-z\,,\,0)\,d\tau-\ln\left(1-\frac{x}{100}\right)\\
\Leftrightarrow\quad & b_w MF_{SD}(x,t)\int_0^t \max_{0<\tau<t}\left(D_w(\tau)-\frac{z}{MF_{SD}(x,t)}\,,\,0\right)\,d\tau=b_w\int_0^t \max_{0<\tau<t}(D_w(\tau)-z\,,\,0)\,d\tau-\ln\left(1-\frac{x}{100}\right)
\end{aligned} \tag{S24}$$

When the external concentration is constant ($C_w(\tau) = C_w$), we can obtain $MF_{SD}(x,t)$ by using the explicit expression of $D_w(t)$ given by (S2). And so, we can define t_z and $t_{z,MF}$ such as:

$$\begin{aligned}
z = C_w\left(1 - e^{-k_d t_z}\right) &\quad \Rightarrow \quad t_z = \frac{-1}{k_d}\ln\left(1 - \frac{z}{C_w}\right) \\
\frac{z}{MF_{SD}(x,t)} = C_w\left(1 - e^{-k_d t_{z,MF}}\right) &\quad \Rightarrow \quad t_{z,MF} = \frac{-1}{k_d}\ln\left(1 - \frac{z}{MF_{SD}(x,t)C_w}\right)
\end{aligned} \tag{S25}$$

Hence, we have:

$$\begin{aligned}
& b_w MF_{SD}(x,t)\int_0^t \max_{0<\tau<t}\left(D_w(\tau) - \frac{z}{MF_{SD}(x,t)}\,,\,0\right)d\tau = b_w\int_0^t \max_{0<\tau<t}\left(D_w(\tau) - z\,,\,0\right)d\tau - \ln\left(1 - \frac{x}{100}\right) \\
\Leftrightarrow \quad & b_w MF_{SD}(x,t)\int_{t_{z,MF}}^t C_w\left(1 - e^{-k_d\tau}\right) - \frac{z}{MF_{SD}(x,t)}\,d\tau = -\ln\left(1 - \frac{x}{100}\right) + b_w\int_{t_z}^t C_w\left(1 - e^{-k_d\tau}\right) - z\,d\tau \\
\Leftrightarrow \quad & b_w MF_{SD}(x,t)\left(\frac{C_w}{k_d}\left(e^{-k_d t} - e^{-k_d t_{z,MF}}\right) + \left(C_w - \frac{z}{MF_{SD}(x,t)}\right)(t - t_{z,MF})\right) = \\
& -\ln\left(1 - \frac{x}{100}\right) + b_w\left(\frac{C_w}{k_d}\left(e^{-k_d t} - e^{-k_d t_z}\right) + (C_w - z)(t - t_z)\right) \\
\Leftrightarrow \quad & MF_{SD}(x,t) = \frac{-\ln\left(1 - \frac{x}{100}\right) + b_w\left(\frac{C_w}{k_d}\left(e^{-k_d t} - e^{-k_d t_z}\right) + (C_w - z)(t - t_z)\right)}{b_w\left(\frac{C_w}{k_d}\left(e^{-k_d t} - e^{-k_d t_{z,MF}}\right) + \left(C_w - \frac{z}{MF_{SD}(x,t)}\right)(t - t_{z,MF})\right)}
\end{aligned} \tag{S26}$$

As for the $LC_{SD}(x,t)$, the expression of $t_{z,MF}$ prevents to have an explicit formulation of $MF_{SD}(x,t)$.

3.3 $MF_{IT}(x,t)$ for model GUTS-RED-IT

Using the definition of the multiplication factor, we have the following formulation for the GUTS-RED-IT model:

$$
\begin{aligned}
& S_{IT}\left(MF_{IT}(x,t)\times C_w(\tau),t\right) = S_{IT}(C_w(\tau),t)\left(1-\frac{x}{100}\right) \\
\Leftrightarrow\quad & e^{-h_b t}\left(1-\frac{1}{1+\left(\frac{\max\limits_{0<\tau<t}\left(MF_{IT}(x,t)\times D_w(\tau)\right)}{m_w}\right)^{-\beta}}\right) = e^{-h_b t}\left(1-\frac{1}{1+\left(\frac{\max\limits_{0<\tau<t}(D_w(\tau))}{m_w}\right)^{-\beta}}\right)\left(1-\frac{x}{100}\right) \\
\Leftrightarrow\quad & 1-\frac{1}{1+MF_{IT}(x,t)^{-\beta}\left(\frac{\max\limits_{0<\tau<t}(D_w(\tau))}{m_w}\right)^{-\beta}} = \left(1-\frac{1}{1+\left(\frac{\max\limits_{0<\tau<t}(D_w(\tau))}{m_w}\right)^{-\beta}}\right)\left(\frac{100-x}{100}\right) \\
\Leftrightarrow\quad & 1-\frac{1}{1+MF_{IT}(x,t)^{-\beta}\left(\frac{\max\limits_{0<\tau<t}(D_w(\tau))}{m_w}\right)^{-\beta}} = \left(\left(\frac{\max\limits_{0<\tau<t}(D_w(\tau))}{m_w}\right)^{-\beta}\frac{1}{1+\left(\frac{\max\limits_{0<\tau<t}(D_w(\tau))}{m_w}\right)^{-\beta}}\right)\left(\frac{100-x}{100}\right) \\
\Leftrightarrow\quad & 1-\frac{1}{1+MF_{IT}(x,t)^{-\beta}\left(\frac{\max\limits_{0<\tau<t}(D_w(\tau))}{m_w}\right)^{-\beta}} = \left(\frac{\max\limits_{0<\tau<t}(D_w(\tau))}{m_w}\right)^{-\beta}\frac{(100-x)}{100+100\left(\frac{\max\limits_{0<\tau<t}(D_w(\tau))}{m_w}\right)^{-\beta}} \\
\Leftrightarrow\quad & 1-\frac{1}{1+MF_{IT}(x,t)^{-\beta}\left(\frac{\max\limits_{0<\tau<t}(D_w(\tau))}{m_w}\right)^{-\beta}} = \frac{100-x}{100+100\left(\frac{\max\limits_{0<\tau<t}(D_w(\tau))}{m_w}\right)^{-\beta}} \\
\Leftrightarrow\quad & \frac{1}{1+MF_{IT}(x,t)^{-\beta}\left(\frac{\max\limits_{0<\tau<t}(D_w(\tau))}{m_w}\right)^{-\beta}} = 1-\frac{100-x}{100+100\left(\frac{\max\limits_{0<\tau<t}(D_w(\tau))}{m_w}\right)^{\beta}} \\
\Leftrightarrow\quad & 1+MF_{IT}(x,t)^{-\beta}\left(\frac{\max\limits_{0<\tau<t}(D_w(\tau))}{m_w}\right)^{-\beta} = \frac{100+100\left(\frac{\max\limits_{0<\tau<t}(D_w(\tau))}{m_w}\right)^{\beta}}{100+100\left(\frac{\max\limits_{0<\tau<t}(D_w(\tau))}{m_w}\right)^{\beta}-(100-x)} \\
\Leftrightarrow\quad & MF_{IT}(x,t)^{-\beta}\left(\frac{\max\limits_{0<\tau<t}(D_w(\tau))}{m_w}\right)^{-\beta} = \frac{100-x}{100+100\left(\frac{\max\limits_{0<\tau<t}(D_w(\tau))}{m_w}\right)^{\beta}-(100-x)} \\
\Leftrightarrow\quad & MF_{IT}(x,t)^{-\beta} = \left(\frac{\max\limits_{0<\tau<t}(D_w(\tau))}{m_w}\right)^{\beta}\frac{100-x}{100+100\left(\frac{\max\limits_{0<\tau<t}(D_w(\tau))}{m_w}\right)^{\beta}-(100-x)} \\
\Leftrightarrow\quad & MF_{IT}(x,t)^{-\beta} = \frac{100-x}{100+x\left(\frac{\max\limits_{0<\tau<t}(D_w(\tau))}{m_w}\right)^{-\beta}} \\
\Leftrightarrow\quad & MF_{IT}(x,t) = \sqrt[\beta]{\frac{100+x\left(\frac{\max\limits_{0<\tau<t}(D_w(\tau))}{m_w}\right)^{-\beta}}{100-x}}
\end{aligned}
\tag{S27}
$$

Therefore, from a GUTS-RED-IT model, solving the toxicokinetic part giving $\max_{0<\tau<t}(D_w(\tau))$ is enough to find any multiplication factor for any x at any t.

When the external concentration is constant (i.e., $C_w(\tau) = C_w$) we get:

$$MF_{IT}(x,t) = \left(\frac{100 + x\left(\frac{C_w\left(1 - e^{-k_d t}\right)}{m_w}\right)^{-\beta}}{100 - x} \right)^{1/\beta} \tag{S28}$$

4 Definition of WAIC and LOO-CV

For more information about information criteria and cross-validation, see [?]. Here we give a brief description to compute the Widely Available/Applicable Information Criterion (WAIC) and the Leave-One-Out Cross-Validation (LOO-CV).

4.1 WAIC: Widely Available/Applicable Information Criterion

The first step is to compute the log pointwise predictive density:

$$lpd = \log \prod_{i=1}^{n} p_{post}(y_i) \Rightarrow \widehat{lpd} = \sum_{i=1}^{n} \log\left(\frac{1}{S}\sum_{s=1}^{S} p(y_i|\theta^S)\right) \tag{S29}$$

Then the variance of the log pointwise predictive density:

$$p_{WAIC} = \sum_{i=1}^{n} \mathbb{V}_{s=1}^{S}(\log p(y_i|\theta^s)) \tag{S30}$$

To finally compute the WAIC as:

$$WAIC = -2(\widehat{lpd} - p_{WAIC}) \tag{S31}$$

4.2 LOO-CV: Leave-One-Out Cross-Validation

Cross-validation is like WAIC, applied several time on a subsample (S-1) drawn from the posterior.

$$\widehat{lpd_{-i}} = \sum_{j=1}^{n} \log\left(\frac{1}{S}\sum_{s=1}^{S} p(y_j|y_{-i};\theta^{is})\right) \tag{S32}$$

Then the first order bias correction

$$\overline{lpd_{-i}} = \frac{1}{n}\sum_{i=1}^{n}\sum_{j=1}^{n} \log\left(\frac{1}{S}\sum_{s=1}^{S} p(y_j|y_{-i};\theta^{is})\right) \tag{S33}$$

To finally compute the LOO-CV as:

$$LOO = -2\left(\widehat{lpd_{-i}} + \widehat{lpd} - \overline{lpd_{-i}}\right) \tag{S34}$$
